# Large Language Models Estimate Fine‐Grained Human Color–Concept Associations

**DOI:** 10.1111/cogs.70219

**Published:** 2026-06-22

**Authors:** Kushin Mukherjee, Ankit Mohapatra, Timothy T. Rogers, Karen B. Schloss

**Affiliations:** ^1^ Department of Psychology Stanford University; ^2^ Wisconsin Institute for Discovery University of Wisconsin‐Madison; ^3^ Department of Computer Science University of Wisconsin‐Madison; ^4^ Department of Psychology University of Wisconsin‐Madison

**Keywords:** Color‐concept associations, Color semantics, Vision‐language models

## Abstract

People reliably associate the meanings of both abstract and concrete words with colors distributed over color space, a phenomenon that influences aspects of visual cognition ranging from object recognition to interpreting information visualizations. Prior research has hypothesized that color–concept associations arise from the cross‐modal statistical structure of experience, but it remains unclear whether natural environments contain such structure or whether learning systems can discover it without strong prior constraints. To address these questions, we investigated whether GPT‐4, a multimodal large language model, can estimate color–concept association ratings that approximate those made by people. We tested 71 colors spanning perceptual color space and a variety of concepts varying in abstractness. GPT‐4 ratings correlated strongly with human ratings across a range of prompting strategies, outperforming prior state‐of‐the‐art methods for automatically estimating color–concept associations from images. In an empirical study assessing people's ability to interpret the meanings of colors in information visualizations, palettes generated from GPT‐4's rating data were not only interpretable but, in some cases, more effective than those based on human ratings. Taken together, our results suggest that high‐order covariance between language and perception, present in web‐scale data, provide sufficient information to learn color–concept associations without initial constraints, and that machine‐derived associations can support the optimization of information visualizations for visual communication.

## 1 Introduction

It is no surprise that people connect the names of familiar objects with their characteristic colors—for instance, strongly associating “carrot” with a particular shade of orange. Recent work suggests, however, that such associations are not limited to characteristic, top‐associated colors of concrete concepts; instead, people can judge association strengths between essentially any concept and any color, regardless of the concept's concreteness or the extent to which it possesses characteristic colors (Jonauskaite & Mohr, 2025; Liu, van Paridon, & Lupyan, 2025; Liu & Lupyan, 2023; Mukherjee, Yin, Sherman, Lessard, & Schloss, 2022; Schoenlein, Campos, Lande, Lessard, & Schloss, 2023). At first, the notion that every concept is associated with every color to some degree, even if that degree is zero, may seem to contradict the classic notion of *color diagnosticity*, defined as the degree to which a color is associated or symptomatic of a particular object (Tanaka & Presnell, 1999). However, as operationalized, color diagnosticity concerns the extent to which colors are readily and consistently listed as features of objects (Tanaka & Presnell, 1999). For example, “fire engine” is color diagnostic because “red” is readily named as a key feature of fire engine, whereas “car” is not color diagnostic because no specific colors are readily named a key feature of car. Yet, both of these notions can be true simultaneously: although people may not think to list a color as a primary feature of a car, they have systematic associations between colors and the concept car, as is the case in the present study. Moreover, considering color–concept associations as distributions over colors has led to advances in understanding how people assign meaning to colors, which cannot be explained by considering only the top‐associated colors for a given concept (Murthy, Griffiths, & Hawkins, 2022; Schloss, 2024; Schloss, Lessard, Walmsley, & Foley, 2018).

This paper considers to what extent contemporary large‐language models and vision‐language models (LLMs/VLMs) can approximate human‐generated color–concept association ratings, with two general goals. The first is pragmatic: color–concept associations influence many aspects of visual cognition, including object recognition (Ostergaard & Davidoff, 1985; Tanaka & Presnell, 1999; Wurm, Legge, Isenberg, & Luebker, 1993), color preferences (Palmer & Schloss, 2010; Schloss & Palmer, 2017; Strauss, Schloss, & Palmer, 2013; Taylor & Franklin, 2012), and visual communication (Jahanian, Keshvari, Vishwanathan, & Allebach, 2017; Lin, Fortuna, Kulkarni, Stone, & Heer, 2013; Mukherjee et al., 2022; Schloss et al., 2018; Setlur & Stone, 2016; Schloss, 2024). Thus, a central aim of color semantics research has been to quantify such associations for large sets of concepts, and several different models have been designed in prior work to explicitly estimate such associations from image/language corpora (Jahanian et al., 2017; Lin et al., 2013; Rathore, Leggon, Lessard, & Schloss, 2020; Setlur & Stone, 2016; Munroe, 2010). If LLMs/VLMs can closely approximate human association ratings, they can provide an efficient, effective, and accessible tool for quantifying such associations across a vast set of possible concepts, which will be useful for future research on color semantics.

The second goal is to evaluate the face validity of one hypothesis about the origins of human color–concept associations: specifically, the proposal that such associations emerge from the mind's statistical learning capabilities (Liu et al., 2025; Schloss, 2018; Schloss, 2024; Schoenlein & Schloss, 2022), which are sensitive to high‐order statistical structure in the environment (Saffran, Aslin, & Newport, 1996; Turk‐Browne, Jungé, & Scholl, 2005; Turk‐Browne, Isola, Scholl, & Treat, 2008). By “high‐order” we mean, not just immediate pairwise correlation between a perceived word or object and a color, but systematic patterns of covariation among words and phrases, observed objects, and the events or scenarios in which these arise. Contemporary models of semantic knowledge acquisition suggest that sensitivity to high‐order covariation can explain many varieties of abstract conceptual representation, such as the deep distinction between living versus nonliving things (Rogers & McClelland, 2004; Rogers et al., 2004) or the biological concept of sex that distinguishes meanings of “bull” and “rooster” from “cow” and “hen” (Mikolov, Sutskever, Chen, Corrado, & Dean, 2013). Perhaps color–concept associations likewise emerge from a learning system sensitive to high‐order patterns of covariation arising in lived experience (Elliot, Maier, Moller, Friedman, & Meinhardt, 2007; Liu et al., 2025; Rathore, Leggon, Lessard, & Schloss, 2020; Schoenlein & Schloss, 2022; Witzel, Valkova, Hansen, & Gegenfurtner, 2011).

While this hypothesis has been advanced in prior work (Liu et al., 2025; Schloss, 2018, 2024; Soriano & Valenzuela, 2009), it has been unclear whether such high‐order structure exists in real‐world language and perceptual experiences, and if it does, whether any learning system is capable of detecting and exploiting it without built‐in constraints designed explicitly to help it do so. Can a learning system that is not purposely engineered to acquire color–concept associations nevertheless come to generate associations that approximate those of humans after training on large data corpora? If so, this would provide evidence that naturalistic corpora contain sufficient information to support learning of color–concept association, and that such associations can emerge as a byproduct of learning in a system driven by other primary objectives.

### 1.1 Using LLMs to study human perceptual knowledge

With the goal of evaluating general learning systems not specifically engineered to generate color–concept associations, we primarily assessed an LLM and VLM from the GPT‐4 family (OpenAI, 2023a), as well as open‐weight variants of transformer‐based LLM/VLM models (Cherti et al., 2023; Yang et al., 2025) on their ability to generate patterns of color–concept associations that approximate those of human participants. Our motivation for choosing this class of models is that many varieties of LLMs—including the BERT (Clark, Luong, Le, & Manning, 2020; Devlin, Chang, Lee, & Toutanova, 2018; Liu et al., 2019; Yang et al., 2019), Llama (Touvron et al., 2023), Flan‐T5 (Chung et al., 2022), and GPT (Brown et al., 2020) families—also produce human‐like behaviors in psychological tasks involving both language (Futrell et al., 2019; Kumar et al., 2022; Lampinen, 2022; Piantadosi & Hill, 2022; Tuckute et al., 2023) and perception (Conwell, Prince, Alvarez, & Konkle, 2023; Mirchandani et al., 2023). Even simpler neural networks can learn some aspects of perceptual structure by training on large image and language corpora; for example, color space coordinates can be decoded from the intermediate representations of autoregressive transformer models (Vaswani et al., 2017) when presented with color terms in natural language (Abdou et al., 2021; Patel & Pavlick, 2021) and vision models trained for object recognition recapitulate the categorical structure underpinning people's representations of colors (de Vries, Akbarinia, Flachot, & Gegenfurtner, 2022).

Given these findings, it may seem that LLMs could, at best, generate associations between concepts and colors that are easily nameable (rather than for all possible perceived colors). However, recent work has shown that the web‐scale datasets used to train contemporary LLMs implicitly encompass an analog of cross‐modal learning (Bubeck et al., 2023; Du et al., 2024; OpenAI, 2023b). Specifically, text‐based languages for rendering visual images (e.g., scalable vector graphics) include standards for referring to highly specific colors, such as hexadecimal‐based color descriptors (hex codes). Such “color tags” are embedded within code used to render images on web pages, which, in turn, is embedded within the natural language describing accompanying text on the page. On this basis, LLMs can complete a remarkable range of tasks that depend on knowledge and/or perception of color—for instance, generating vector graphics of animals and scenes using text descriptions as input (Bubeck et al., 2023) or providing judgments about perceptual similarity (Marjieh et al., 2023).

More generally, recent work has shown that multimodal VLMs like GPT‐4 and Google's Gemini models (Team et al., 2024) learn representations of visual concepts that are comparable to people's conceptual representations (Du et al., 2025; Xu et al., 2025), which themselves are interpretable by established standards (Hebart et al., 2023). These studies found that *multimodal* models were more strongly aligned with both behavioral (Du et al., 2025; Xu et al., 2025) and neural (Du et al., 2025) measurements of human concepts relative to language‐only models. This prior work provides converging evidence that generalized mechanisms for learning visual concept representations might enable multimodal VLMs to learn color–concept associations, even for abstract concepts, from complex patterns of covariation between language and textual signifiers of color. However, given that these prior studies evaluated datasets that are based on concept‐to‐concept psycholinguistics norms or concept‐concept similarity ratings, an open question remains as to whether these models can capture the precise distribution of color‐concept associations when the colors are presented in finer granularity than basic color terms commonly used in natural language (e.g., as hex codes or color patches).

### 1.2 Automatic estimation of color–concept associations

Prior work on automatically quantifying people's color–concept associations have relied on engineering models and systems whose main goal is to automatically generate color–concept association values for a queried concept (Bartram, Patra, & Stone, 2017; Lin et al., 2013; Rathore et al., 2020; Setlur & Stone, 2016; Van De Weijer, Schmid, Verbeek, & Larlus, 2009). In doing so, this work aimed to automate the effective use of color in information visualization. These approaches stand in contrast to models like GPT‐4, which are primarily trained on next‐word prediction and various general post‐training regimes, and do not include special machinery to learn color–concept associations in particular. The non‐LLM class of approaches have leveraged visual knowledge latent in large‐scale language databases, image databases, or a combination of both. Language‐based techniques exploited co‐occurrences between single words and color information in large linguistic corpora (Bartram et al., 2017; Havasi, Speer, & Holmgren, 2010; Lindner, Li, Bonnier, & Süsstrunk, 2012; Lin et al., 2013; Rathore et al., 2020). For example, Havasi et al. (2010) used a semantic network, ConceptNet (Liu & Singh, 2004; Speer, Chin, & Havasi, 2017), to find color terms associated with concept words, and mapped these color terms to a perceptual space using the XKCD color‐naming dataset (Munroe, 2010). While this method allowed them to find “best” associated color for a concept, their method did not estimate a distribution over color space. Subsequent studies used language corpora to map concepts to basic color terms, then linked those color terms to colored patches using information in labeled image databases (Setlur & Stone, 2016), following a method established by Lin et al. (2013). Other approaches have also combined information latent in natural images and language corpora using topic modeling approaches (Jahanian et al., 2017) and color distributions associated with words to establish the semantic relatedness between concepts (Guilbeault et al., 2020; Mukherjee, Schloss, Lessard, Gleicher, & Rogers, 2022; Mukherjee, Rogers, Lessard, Gleicher, & Schloss, 2021). When estimating color–concept associations from images, it is necessary to specify which pixel inputs should “count” toward an association between a given color and concept. For example, when evaluating images of blueberries to estimate associations between colors and the concept *blueberry*, should an algorithm only consider the exact pixel colors in the image, or should there be some “tolerance” around the pixel colors? Rathore et al. (2020) found that the method that best matched human judgments used a tolerance that scaled in size along perceptual dimensions of color and used *category extrapolation* to spread to all colors that shared the same color category as the pixel input (e.g., observing a grayish blue pixel in an image of blueberries counted for all colors categorized as *blue*). Bypassing the issue of pixel tolerance, Hu, Ye, Chen, van Kaick, and Huang (2023) finetuned a pretrained neural network model to predict pixel colors of grayscale images (colorization) and used it to estimate color–concept associations—a method particularly effective for capturing the overall scale of association values.

Despite these advances, several limitations preclude using such methods to estimate color–concept association distributions for *any* queried concept. Language‐based approaches typically return the top associate or optimal color but do not provide association distributions over color space (Havasi et al., 2010; Setlur & Stone, 2016). The design of effective visualizations depends critically upon such distributions because a concept's strongest associate may not be the most interpretable color depending on the context—the other concepts and colors appearing in the display (Mukherjee et al., 2022; Schloss et al., 2018). Image‐based approaches address this limitation by generating distributions of associations, but rely on databases like Google Images that in turn depend on varied and ever‐changing interfaces and levels of access. Moreover, implementing specialized computer vision algorithms often takes time and domain‐specific knowledge. In contrast, contemporary artificial intelligence (AI) technologies have the potential to quickly return color–concept association distributions for any concept in a relatively easy‐to‐use manner.

### 1.3 Overview of the present work

In the present study, we first investigated the extent to which GPT‐4, an LLM that successfully captures human similarity judgments for a variety of perceptual features, including color, sound, and touch (Marjieh et al., 2023), could approximate the distribution of color–concept associations for colors finely sampled across color space (Experiment 1). We then compared the results from GPT‐4 to those of a newer vision‐language model (GPT‐4V), which can take input from both text and images and thus can generate color–concept ratings for bitmap images of color patches, or from hex codes and images together (Experiment 2A). In both cases, we compared model and human behaviors, assessing whether models can produce variable rating strengths for any color–concept pair, and if so, whether such ratings correlate with those produced by human participants for the same items (Fei et al., 2024; Kewenig et al., 2023). Answers to these questions address both the pragmatic and theory‐based aims of the work.

Experiments 1 and 2A focused on GPT‐4 as a frontier language model that is highly performant on the majority of machine learning benchmarks. Given the black‐box nature of these models, however, it is difficult to attribute differences in performance between different model variants to specific model properties, such as whether the model was trained on both text and images. To address this issue, we conducted Experiment 2B to replicate core analyses from Experiment 2A using two families of open‐weight models—Qwen‐3 and OpenCLIP. This experiment further allowed us to test the generalizability of our findings beyond a single model family.

With evidence from Experiments 1 and 2 that LLMs/VLMs are performant at estimating human color–concept associations, we next investigated how LLMs/VLMs performed relative to other contemporary methods for automatically estimating color–concept associations (Experiment 3). We compared results from Experiments 1 and 2 to the performance of two recent bespoke models designed explicitly to estimate color–concept associations (Hu, Ye, Chen, van Kaick, & Huang, 2023; Rathore et al., 2020). From a practical standpoint, this comparison tested whether contemporary AI can be as or more effective than state‐of‐the‐art computational models in generating such estimations for myriad applications. From a theoretical standpoint, Experiment 3 evaluated how the color–concept associations that emerge as a byproduct of learning in contemporary AI compare to those arising within a system specifically engineered for this purpose.

Experiment 3 established that VLMs produced color–concept associations that correlated with human judgments better than prior approaches to automatically estimating color–concept associations, but VLM associations are still nonperfect estimates of human associations (Experiment 2A). In Experiment 4, we sought to evaluate whether VLM estimates were *good enough* to predict human behavior in a cognitive task that relies on color–concept associations. Specifically, we evaluated whether human participants can make use of AI‐generated color–concept associations when inferring the meanings of colors in data visualizations. Prior work has shown that if color palettes for a data visualization are designed to maximally discriminate possible concepts on the basis of their (human‐generated) color associations, people can reliably infer the intended meanings without a color legend (Mukherjee et al., 2022; Schloss et al., 2021; Schloss et al., 2018). Likewise, people are slower and more error‐prone to interpret color meanings if the palette does not differentiate potential meanings, even when a legend is present (Lin et al., 2013). We tested whether visualizations optimized on the basis of AI‐generated color–concept associations were as effective in guiding human interpretations of color meaning in data visualizations as those based on human‐generated associations. If so, that would suggest that machine estimates of human color–concept associations are good enough to predict judgments that rely on such associations, at least for the concepts and task we assessed.

## 2 Experiment 1

**Fig. 1:**
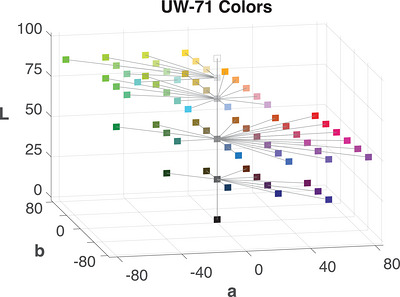
Colors in the UW–71 color library plotted in CIELAB space (figure adapted from Mukherjee et al., 2022).

Experiment 1 assessed whether GPT‐4 can produce color–concept associations correlated with those produced by people when given prompts similar to human instructions and using hex codes to indicate each color. The concepts spanned a wide variety of semantic domains, including concrete and abstract items, items with characteristic colors, and items without strong color associates (Table 1). For every concept, we measured association strength with each of the UW‐71 colors, a color set that systematically samples colors across CIELAB space (Fig. 1). For human judgments, we used data collected in previous work (Mukherjee et al., 2022; Mukherjee et al., 2022; Mukherjee et al., 2021) asking participants to judge associations between concepts and visually displayed color patches. For GPT‐4, we collected new judgments using a prompt derived from the human task instructions but using hex codes in place of color patches. We then assessed whether (a) GPT‐4 would generate interpretable, numerical rating responses for each color–concept pair and (b) if so, how well these ratings correlated with mean human ratings across all 71 colors for each concept.

**Table 1:** Categories of concepts tested in this study (five concepts per category), and corresponding number of participants (*n*) for each category

Category	Concepts	*n*
Activities	Driving, Eating, Sleeping, Leisure, Working	52
Animals	Bear, Bird, Lion, Frog, Fish	45
Automobiles	Airplane, Car, Boat, Truck, Train	51
Clothes	Dress, Pants, Shirt, Socks, Shoes	48
Directions	Above, Below, Beside, Near, Far	44
Emotions	Angry, Disgust, Fearful, Happy, Sad	50
Fruits	Blueberry, Lemon, Mango, Strawberry, Watermelon	46
Fruits2	Apple, Banana, Cherry, Grape, Peach	49
Properties	Comfort, Efficiency, Reliability, Safety, Speed	50
Scenes	Beach, Field, Ocean, Sky, Sunset	45
Times of Day	Dawn, Day, Dusk, Noon, Night	46
Values	Evil, Greed, Justice, Love, Peace	50
Vegetables	Carrot, Celery, Corn, Eggplant, Mushroom	52
Weather	Blizzard, Drought, Hurricane, Lightning, Sandstorm	52

### 2.1 Methods

In this section, we first describe the procedure for collecting color–concept associations from humans (previously reported in Mukherjee et al., 2022 and Mukherjee et al., 2021), followed by our new procedure for estimating color–concept associations from GPT‐4.^1^ Our general approach was to present pairs of colors and concepts and have both humans and models provide ratings on a scale from “not at all” to “very much,” indicating how much they associated the given concept with the given color. Given the large number of color–concept pairs evaluated in this experiment, we use a ratings task because ratings are efficient, albeit less sensitive than other measures such as two‐alternative forced choice (Palmer, Schloss, & Sammartino, 2013).

#### 2.1.1 Methods for human data

**Participants**. The dataset was collected from 720 participants across three studies ($M_{age}=$ 19.30, self‐reported through free‐response as 415 women, 302 men, 1 nonbinary, 1 self‐reported as “other”) who participated for extra credit in their undergraduate psychology course at the University of Wisconsin‐Madison, previously reported by Mukherjee et al. (2022, 2021). All participants provided informed consent and the UW‐Madison IRB approved the protocol. Across studies, a total of 63 participants were excluded due to atypical color‐vision as assessed through “no” responses to one of two self‐report questions or failure to correctly identify the number in more than one digital Ishihara plate from a total of six plates.

**Design and displays**. Each trial presented a single concept appearing as a written word and a single color patch presented as a colored square. The concepts included five items drawn from each of 14 categories selected to broadly vary semantic domain (e.g., clothes, time of day, spatial relations, etc.), concreteness (e.g., “shirt” vs. “love”), and specificity (e.g., “banana” vs. “beside”; see Table 1). Each participant was assigned one of the 14 concept categories and judged each of the five concepts within that category in a random order.

The colors were all 71 colors in the UW‐71 color library, which includes 58 colors sampled uniformly in CIELAB space (color distance ΔE = 25) (Rathore et al., 2020), plus 13 additional colors sampled at higher lightness (L* = 88) to include more “typical” yellows excluded from the grid sampling (Mukherjee et al., 2022) (Fig. 1). The word and color appeared against a dark gray background (CIE Illuminant D65, x = 0.3127, y = 0.3290, Y = 10 cd/$m^{2}$) to enable perception of all lightness levels in the UW‐71 colors. Participants judged the colors online on their own devices so the color coordinates were estimates of “true” CIELAB color coordinates (see Table J.1 in Supplementary Materials)—a common approach among studies of color in information visualization, where findings must tolerate natural variation in display characteristics (Gramazio, Laidlaw, & Schloss, 2017; Mukherjee et al., 2022; Stone, Szafir, & Setlur, 2014; Szafir, 2018; Schoenlein et al., 2023). During the task, the participants judged each color for each of their five assigned concepts in a random order for each concept.

**Procedure**. The participants were instructed that they would be presented with each of five concepts along with each of 71 colors, and they would be asked to rate their association between the color–concept pair on a scale from “not at all” to “very much.” Before beginning the rating trials, they completed an anchoring task so they knew what associating “not at all” and “very much” meant in the context of these colors and concepts (Palmer et al., 2013). During anchoring, participants viewed a screen showing all five concepts and all 71 colors and were asked to think about which color they most and least associated with each concept and to plan to rate those concepts near the endpoints of the rating scale during the experiment. They were also asked to use the full range of the scale.

**Fig. 2:**
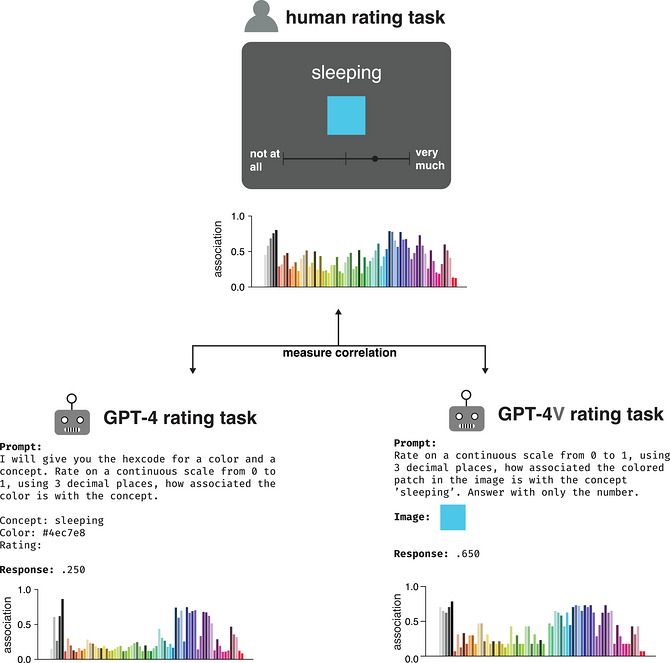
Example trial from the human color–concept association rating task (top) and GPT‐4 rating task for the same color–concept pair using hexadecimal codes (bottom left) and colored patches (bottom right). Bar graphs correspond to average human and GPT‐4 color–concept association ratings for the concept “bird” over the entire UW‐71 color library.

Next, on each trial, participants viewed a single word appearing above a colored patch and used the slider to indicate how much they associated the named concept with the perceived color. After adjusting the slider, they clicked a “continue” button to record their responses (Fig. 2). Using these data, we computed the mean association strength for a given color and concept across participants who rated that pair. The resulting values were the human color–concept association strengths used for the analyses in Experiments 1–3.

### 2.2 Methods for GPT‐4

For GPT‐4, we adapted the color–concept association rating procedure conducted with human participants. We began by assigning a “system” level prompt instructing the model to role‐play as “an expert on color–concept associations.” We then evaluated three prompting methods in their ability to best capture people's association ratings.

**Prompt method 1.1: No anchoring**. This condition queried the model for association ratings given only a single concept and color, without additional anchoring information indicating the range of colors or concepts to be evaluated and with no information about the background color.

The prompt began with the following two sentences describing the task:

“I will give you the hex code for a color and a concept word.
Rate on a continuous scale from 0 to 1, using 3 decimal places, how
associated the color is with the concept.”




Next, we added a sentence indicating the start of a rating trial, followed by three lines: one indicating the concept, one indicating the hex code for the UW‐71 color, and a blank field for the rating with text asking the model to *only* answer with a number. Here is an example of what this procedure was like for the concept “apple” and the color hex code “#FFFFFF”:

Let's do the rating task ‐
Concept: ‘apple'
Color: #FFFFFF
Answer with only the number:




Each rating was obtained using this prompt structure, swapping just the concept and color depending on the pair being rated on a given trial. The temperature was set to 0 to produce deterministic outputs (i.e., multiple prompts for the same color–concept pair would result in the same output). Each color–concept pair was rated once (71 colors × 70 concepts = 4970 total ratings). The model was consistent in providing correctly formatted responses for all prompts in the current and follow‐up experiments in the present work.

**Prompt method 1.2: Anchoring**. This condition provided an analog of the anchoring procedure used with humans: the prompt included all the possible colors that the model could be asked to rate. Specifically, each trial began with two additional sentences as follows (using “apple” as an example):

The concept is ‘apple'.
Before rating, here's the set of all the colors all_color_hexcodes.
Think of which color you associate most with ‘apple.'
That color should get a rating of 1.
Now think of which color you associated least with ‘apple.'
That color should get a rating of 0. Now let's do the rating task.




Here, all_color_hexcodes refers to a comma‐separated list of all 71 color hex codes for the UW‐71 color library. Like prompt method 1.1, the temperature parameter was set to 0, and we collected a total of 4970 ratings.

**Prompt method 1.3: Multiple ratings**. In the first two conditions, GPT‐4 generated deterministic responses (temperature = 0); however, human color–concept associations are stochastic, which is why we compute average ratings across many participants for each pair. To assess whether a mean over stochastic samples provides a better estimate of human ratings, we collected 10 judgments from GPT‐4 using a temperature of 1 (without anchoring) and computed the mean rating for each color–concept pair.

### 2.3 Results and discussion

#### 2.3.1 GPT‐4's color–concept association ratings approximate those of humans

In comparing human color–concept association ratings with those generated by models, we used Pearson correlations between human and model ratings as the key measure of interest. This is because it has been shown that *relative* differences in  associations between colors and concepts are a key driver of the meanings people ascribe to colors (Mukherjee et al., 2022; Schloss et al., 2018; Schoenlein et al., 2023; Schloss et al., 2021). We also report average root mean squared error (RMSE) scores for the different prompting methods presented in Supplementary Materials.

**Fig. 3:**
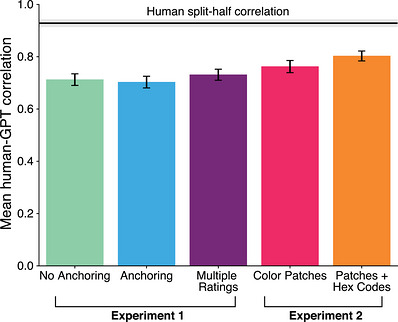
Average correlations between human color–concept association ratings and GPT‐4 association ratings for each of the prompting methods in Experiments 1 and 2. Errorbars represent the ± standard errors of the mean. The solid black line represents the mean human split‐half correlation with the errorband representing the standard error.

**Fig. 4:**
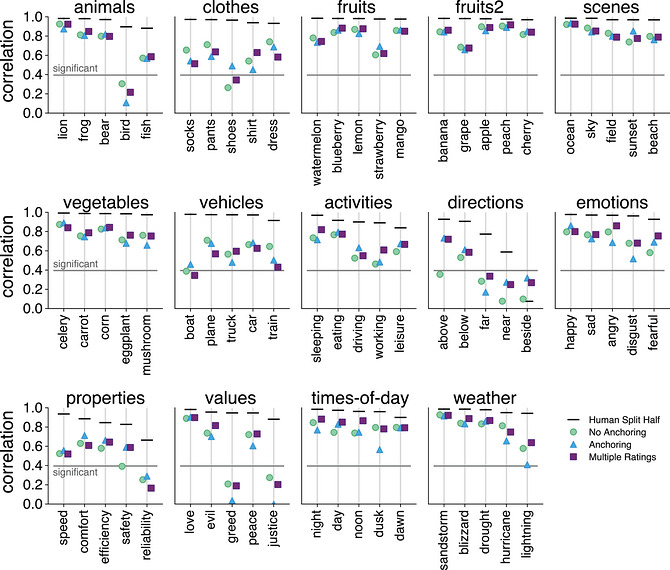
Correlations between average human color–concept association ratings and predicted ratings from GPT‐4 from Experiment 1 for each of 70 concepts. Black lines above each concept correspond to the average human split‐half reliability for that concept. Gray lines in each graph correspond to correlations we would expect by chance after correcting for multiple comparisons. “significant” indicates the correlation value that would be statistically significant after Bonferonni corrections for multiple tests.

Given that even human participants do not perfectly agree with each other in terms of color–concept association ratings, we first sought to establish a meaningful performance “ceiling” based on human ratings data using split‐half correlations. For each concept, we randomly divided the human participants into two groups, calculated the mean association for each color within the two groups, then computed the correlation over association ratings between the two groups for that concept. The Spearman–Brown correction to this correlation value constitutes the split‐half reliability for the concept for a given split of the data. We repeated this procedure 500 times, randomly splitting the data on each iteration, and averaged the split‐half correlations to obtain a mean split‐half correlation for each concept. Fig. 3 shows the mean split half‐correlation averaged across concepts as a solid black line with the gray errorband representing the standard error of the mean and Fig. 4 shows the per‐concept split‐half correlations as black horizontal lines above each concept.

Next, for each concept, we computed the correlation between GPT‐4 estimates and mean human association ratings across colors for each of the three prompt methods. We refer to the correlation between model and human ratings as the *alignment* between these two agent types.

***No anchoring***. Ratings from the no‐anchoring method correlated significantly with human ratings, yielding an average of r(69) =  .71 across the 70 concepts. The correlation varied substantially across concepts (max r(69) =  .93, min r(69) =  .08). The gray lines in Fig. 4 indicate the r value above which a correlation was considered statistically significant after Bonferroni‐correction for 70 correlations corresponding to the 70 concepts (critical α=0.0007). Correlations exceeded this r value for 59 out of 70 concepts with the no‐anchoring strategy. A paired samples t‐test over concepts indicated that GPT‐4 (no anchoring) correlations were significantly lower than the split‐half correlations (mean r(69) =  .93), t(69) = 10.67, p <.001). Thus, GPT‐4 generates color–concept association ratings that are strongly correlated with human ratings, but which do not reach the level of human split‐half reliability.

***Anchoring***. The anchoring method yielded comparable results (mean r(69) = .70) to the no‐anchoring method, with no significant difference in correlation strengths between methods (paired t(69) = 0.49, p = .62). Out of 70 concepts, 63 concepts showed statistically significant correlations after applying the Bonferroni correction (comparable to the 59 concepts under the no‐anchoring method). The anchoring method also performed below the human split‐half reliability baseline (paired t(69) = 9.12, p <.001). Thus, the additional context provided to the model via the anchoring task did not reliably improve its estimates over the no‐anchoring method.

***Multiple ratings***. The multiple ratings method showed the strongest mean correlations with the human ratings (r(69) =  .73), significantly higher than the no‐anchoring method (paired t(69) = 2.29, p <.05) but not different from the anchoring method (paired t(69) = 1.65, p =  .10). Of the 70 concepts, 61 showed statistically significant correlations when using this strategy (after Bonferroni correction), which was lower than the number of concepts for the anchoring method but higher than the number of concepts in the no‐anchoring method. Here too, performance fell short of the human split‐half reliability baseline (t(69) = 9.36, p <.001). Thus, querying the model multiple times led to a modest increase in alignment of association ratings, but it did not overwhelmingly improve upon the previous methods.

We considered that performance for the multiple ratings method may have further improved had we included more than 10 ratings per color–concept pair. However, human–GPT alignment leveled off at around five ratings, suggesting more ratings would not have helped (see Section B in Supplementary Materials).

#### 2.3.2 When do GPT‐4 ratings align with human ratings?

Fig. 4 shows that, within each broad semantic category, GPT‐4 ratings align strongly with human ratings for some items (e.g., “lion,” “peach,” “above,” “love”) but not others (e.g., “bird,” “shoes,” “near,” “greed”). We next considered whether the *specificity* of a concept's color association distribution may play a role in explaining this item‐wise variability. Specificity is the extent to which some colors are strongly associated with a concept over others, which is captured by the “peakiness” of the color–concept association distribution for a given concept (Mukherjee et al., 2022).

To this end, for each concept, we computed the specificity of the human color–concept associations as the inverse of the Shannon entropy of a concept's associations over the full set of UW‐71 colors (following Mukherjee et al., 2022). Entropy is high when the distribution is relatively flat across colors, so inverse entropy captures the “peakiness” of the distribution. We then normalized this measure to the 0–1 range across all concepts and computed the log to linearize the data. This specificity metric captures the degree to which an association distribution deviates from a purely uniform distribution, with higher values meaning higher specificity.

**Fig. 5:**
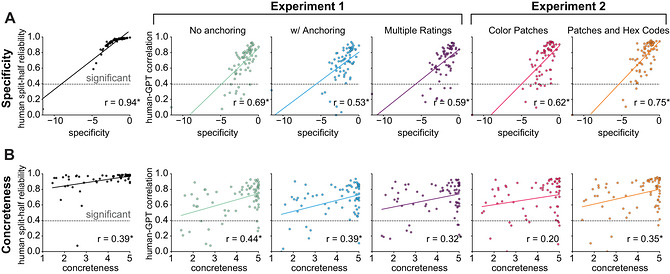
(A) Relationship between the specificity of concepts’ color–concept associations and GPT‐4's ability to accurately predict associations. Each point represents a different concept. (B) Relationship between the concepts’ concreteness and GPT‐4's ability to accurately predict associations. The first column in (A) and (B) reflect human split‐half correlations for color–concept associations as a function of specificity (top) and concreteness (bottom). “Significant” indicates the correlation value that would be statistically significant after Bonferonni corrections for multiple tests.

Concept specificity correlated significantly with human split‐half correlations (Fig. 5A, leftmost panel; r(69) =  .94, p <  .001), indicating that human ratings were more reliable across participants for concepts with more specific distributions of color associations. Specificity also strongly predicted human–GPT correlations across all 70 concepts for all three prompting strategies (Fig. 5A, Experiment 1). As specificity increased, so did human–GPT correlations. Thus, GPT‐4 estimates were less aligned with human estimates for concepts that had lower specificity, and concepts with lower specificity also had lower intersubject agreement. Thus, GPT‐4 showed lower alignment for concepts where human intersubject agreement was also low.

**Fig. 6:**
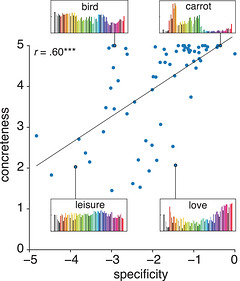
The relationship between specificity and concreteness for the set of 70 concepts. The color–concept association distributions for each of the concepts bird, carrot, leisure, and love are plotted on a scale from 0 to 1.

We also considered the possibility that GPT‐4 might be worse at estimating associations for concepts that are abstract, given that abstract concepts do not possess observable colors that would be reflected in the models’ training corpora. To test this hypothesis, we used word concreteness ratings collected by Brysbaert et al. (2014) and investigated whether a concept's concreteness predicted human–model alignment. Concreteness was moderately correlated with specificity (r(68) =  .60, p <  .001) (Fig. 6), a phenomenon noted by Guilbeault et al. (2020) for a smaller set of concepts, but there were cases that distinguished these two constructs. For example, “bird” and “carrot” are both concrete but “carrot” has far greater specificity. And, “leisure” and “love” are both abstract, but “love” has far greater specificity (see Fig. 6). In isolation, concreteness correlated significantly with both human split‐half correlations and human–model alignment for all three prompting methods (see Fig. 5B). But, when multiple linear regression was used to predict human–model alignment from both concreteness and specificity, only specificity explained significant variance (Table 2). Thus, human–model alignment is better understood in terms of specificity than concreteness.

**Table 2:** Results from linear regression models predicting human–GPT color–concept association rating correlations from concreteness and specificity

Prompt strategy	Predictor	β	*SE*	t	p
No anchoring	Intercept	1.015	0.175	5.80	<.001
	Concreteness	0.04	0.04	1.20	.24
	Specificity	0.14	0.03	5.51	<.001
Anchoring	Intercept	0.95	0.19	4.95	<.001
	Concreteness	0.04	0.04	1.01	.31
	Specificity	0.11	0.04	3.99	<.001
Multiple ratings	Intercept	1.21	0.19	6.27	<.001
	Concreteness	0.002	0.04	0.06	.95
	Specificity	0.14	0.04	4.94	<.001
Color patches	Intercept	1.44	0.24	6.10	<.001
	Concreteness	−0.02	0.05	−0.37	.71
	Specificity	0.19	0.04	5.38	<.001
Color patches + Hex codes	Intercept	1.42	0.21	6.62	<.001
	Concreteness	0.02	0.04	0.37	.72
	Specificity	0.19	0.03	6.02	<.001

*Summary of Experiment 1*. Experiment 1 showed that GPT‐4 can generate color–concept associations when using hex code representations of colors. All three prompting methods produced ratings that correlated significantly with human judgments for a large majority of concepts, differing only slightly in their alignment with human ratings, and with no single method consistently outperforming the others across all concepts. Moreover, the strength of human–GPT‐4 alignment was related to the specificity of the (human) color–concept association distribution, with poor alignment arising when these distributions were relatively flat, and where human intersubject agreement was also lowest. Although concreteness does predict human–model alignment, this relationship appears to reflect the same specificity effect, as more concrete items tended to have more specific distributions. When both measures were included in a regression predicting alignment strength, only specificity explained unique variance.

Together, the results suggest that (a) GPT‐4 may provide a useful basis for estimating human color–concept associations at scale, especially in cases where such associations have high specificity (and thus are highly reliable in human data); and (b) color–concept associations correlated with human decisions can emerge as a byproduct of learning in contemporary AI systems.

## 3 Experiment 2

Experiment 1 used hex codes as a proxy for visual perception when generating GPT‐4 judgments. The advent of capable VLMs provides an alternative means of testing whether color–concept associations can be learned from environmental structure. VLMs can accept both visual and text input, and in addition to the proxies for visual structure that arise in text‐only models, they learn from web‐based corpora that include digital photographs, as well as text and text‐based programming and rendering languages. Thus, such models are capable of assimilating a much broader variety of visual and cross‐modal structure. Recent work by Xu et al. (2025) found that human conceptual representations align better with those of VLMs than pure LLMs, especially for more “visual,” imageable, and concrete items—though such differences can be difficult to interpret as VLMs and LLMs can differ in many respects beyond just the inclusion of photographs as training materials. Experiments 2 evaluated whether estimating color–concept association ratings using both state‐of‐the‐art, proprietary VLMs (Experiment 2A) and analogous open‐weight VLMs (Experiment 2B), which are more transparent, would lead to distributions that were overall more correlated with human ratings than those from pure language models.

## 4 Experiment 2A

Experiment 2A assessed whether color–concept associations generated from GPT‐4V (a vision‐language model variant) are more correlated with human ratings relative to those estimated from GPT‐4 (a language‐only variant). We used the same human data for comparison as in Experiment 1.

### 4.1 Methods

To prompt GPT‐4V, we used the no‐anchoring prompt method from Experiment 1 because all three methods yielded similar outcomes in Experiment 1, and this method was the simplest. Our approach was the same as in Experiment 1, except that we tried two new ways of presenting colors to GPT‐4V: color patch only and color patch and hex codes.

**Prompt method 2.1: Color patches only**. In this condition, the input prompt consisted of a text‐based sentence describing the task followed by a PNG image input consisting of the colored patch. As an example, the prompt for *apple* was:

Rate on a continuous scale from 0 to 1, using 3 decimal places, how
associated the colored patch in the image is with the concept ‘apple'.
Answer with only the number:




This was followed by a 50px×50px image of a colored patch corresponding to one of the UW‐71 colors. The temperature was set to 0 to avoid stochastic outputs. Each color–concept pair was rated once (71 colors × 70 concepts = 4970 total ratings).

**Prompt method 2.2: Color patches and hex codes**. Since VLMs learn from both images and from visual rendering languages, the combination of these two channels may provide more information than either alone. The second condition thus prompted the model with both the color patch in the visual channel (exactly as in the preceding strategy) and the corresponding hex code in the language channel.

For instance, the example prompt using “apple” was:

Rate on a continuous scale from 0 to 1, using 3 decimal places, how
associated the colored patch in the image is with the concept ‘apple'.
The hex‐code for the colored patch is #FFFFFF.
Answer with only the number:




The temperature was set to 0. Each color–concept pair was rated once for a total of 4970 ratings.

### 4.2 Results and discussion

**Fig. 7:**
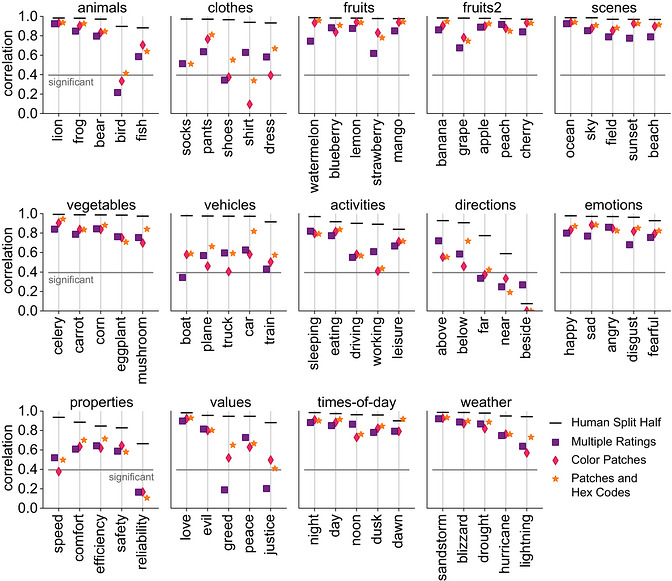
Correlations between average human color–concept association ratings and predicted ratings from GPT‐4V from Experiment 2 for each of 70 concepts. Black lines above each concept correspond to the average human split‐half reliability for that concept. Gray lines in each graph correspond to correlations we would expect by chance after correcting for multiple comparisons. “Significant” indicates the correlation value that would be statistically significant after Bonferonni corrections for multiple tests.

Fig. 3 shows the mean human–model alignment across concepts for each prompting method, while Fig. 7 shows the alignment separately for each concept. While all the responses generated by GPT‐4V were valid (i.e., floats between 0 and 1), the model rated the associations between the concept “beside” and all colors as 0. Due to this lack of variance in ratings for this concept, we exclude it from all correlation computations reported below. For color‐patches only, performance was comparable to Experiment 1: we observed statistically reliable human–model correlations for 59 out of 70 concepts (after applying the Bonferroni correction as in Experiment 1), with a mean r =  .76 across concepts— significantly lower than human–human reliability (mean r(69) =  .93, paired t(68) = 7.89, p <.001) and not different from the best performing text‐only method (multiple ratings mean r(69) =  .73; paired t(68) = 0.02, p =  .98). Thus, use of color patches in place of hex codes in the GPT‐4V model did not produce better estimates of people's color–concept associations.

Prompting GPT‐4V with both color patches and hex codes, however, produced markedly better performance. Of the 70 concepts, 65 showed statistically significant positive correlations after correcting for multiple comparisons. While the correlations across concepts (mean r(69) =  .80) still fell short of the human consistency baseline (mean r(69) =  .93; paired t(68) = 8.83, p <.001), this method did outperform the best text‐only method (multiple ratings with GPT‐4) (paired t(68) = 3.75, p <.001) and the color patches only method with GPT‐4V (paired t(68)  = 3.19, p <.01).

Similar to Experiment 1, concept specificity and concreteness each individually predicted human–GPT alignment across concepts (Fig. 5; for specificity, r(68) =  .75, p <  .001 for concreteness, r(68) =  .35, p <  .05). When both factors were entered into a multiple linear regression model, specificity was again a significant predictor, while concreteness was not, a result observed for both prompting strategies (see Table 2 for model results).

*Summary of Experiment 2A*. Experiment 2A suggests that the potentially richer expression of visual structure arising via incorporation of images in VLMs does not, in itself, lead to improved human–model alignment for color–concept association. Yet, by providing a second channel for encoding visual structure, hex codes in addition to images, such models provide two complementary modes for specifying the critical visual information (i.e., the perceived color). This redundancy across channels appeared to further improve the alignment between human and model ratings.

## 5 Experiment 2B

In frontier language models like GPT‐4 and GPT‐4V, model weights and information about model architecture and training are proprietary, making it difficult to understand what causes differences like those observed in Experiment 2A. The better performance of the VLM prompted with hex codes and images may arise because color–concept associations are better reflected in the statistical structure apparent in multimodal learning, or because of differences in model architectures, corpora, or training procedures. Parallel analyses of open‐source models can help to adjudicate such questions, but introduce different constraints: open‐source architectures comparable to the best frontier models require compute infrastructure inaccessible to most researchers, and models small enough to run on consumer‐grade hardware are typically much less performant on most tasks. To balance these challenges, Experiment 2B replicated the key contrast between models that can accept only language input versus those that can accept multimodal input using a set of models that were runnable on consumer‐grade graphical processing units (GPUs). By virtue of their smaller size, estimates produced by such models are likely to be less correlated with human judgments; the central question is whether the inclusion of multimodal information nevertheless improves alignment within open‐source LLMs/VLMs as it did with the frontier models. Thus, we evaluated two recent state‐of‐the‐art open‐weight models, Qwen‐3‐Instruct and Qwen‐Instruct‐VL (Yang et al., 2025) (henceforth, Qwen‐3 and Qwen‐3‐VL, respectively), using the 4 billion parameter variants to control for model size and to ensure that multimodal input was the salient distinguishing factor between models.

Finally, to assess whether human–model alignment depends on the particular capabilities of LLMs/VLMs, we additionally evaluated OpenCLIP, an open‐weight model based on the multimodal CLIP architecture (Cherti et al., 2023). Unlike VLMs, CLIP models cannot generate text; but like VLMs, they are trained on web‐scale multimodal corpora and can embed both image and text input into modality‐specific spaces. This comparison thus assesses whether good alignment can obtain within a somewhat simpler multimodal transformer framework. Here, we used the ViT‐B‐32 vision encoder variant that was trained on the proprietary “openai” corpus. This vision encoder has been shown to be generally performant on a range of computer vision tasks, often performing comparably to people (Amir, Gandelsman, Bagon, & Dekel, 2021; Dosovitskiy et al., 2020).

### 5.1 Methods

Simulations with the Qwen models used the same prompts as Experiment 1 (no anchoring) and Experiment 2A. Since OpenCLIP cannot generate text like VLMs, we computed the cosine similarity between image embeddings of the color patches and text embeddings of the concepts as a measure of association strength. Higher cosine similarity indicates stronger associations. Here, we focused on the simplest text prompt that did not include extraneous information—just the concept word itself. We report performance under two alternative prompt templates in Supplementary Materials Section D. To put the ratings on the same scale as human ratings, we min‐max normalized the cosine similarities across all concepts and colors such that the highest similarity observed was set to 1, the lowest similarity to 0, and all other ratings falling between the two. To compare the model ratings to that of humans, we computed the Pearson correlation between these normalized model ratings and the mean human ratings for each color–concept pair.

### 5.2 Results and discussion

**Fig. 8:**
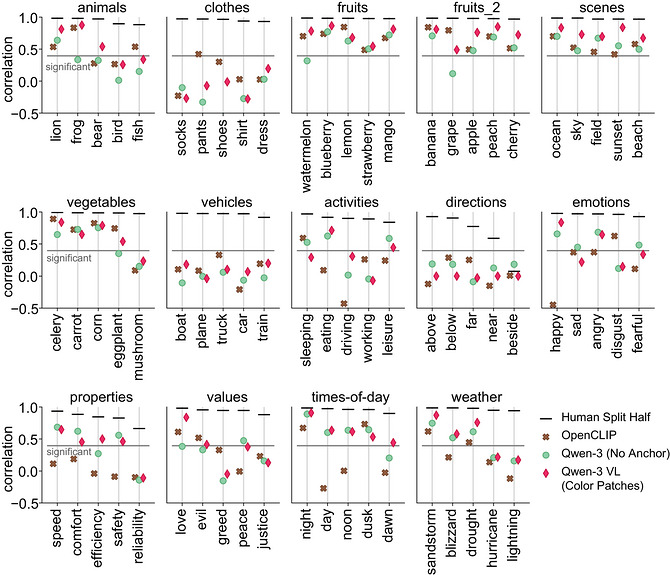
Correlations between average human color–concept association ratings and predicted ratings from OpenCLIP, Qwen‐3 (LLM), and Qwen‐3 VL (VLM) from Experiment 2B for each of 70 concepts. Black lines above each concept correspond to the average human split‐half reliability for that concept. Gray lines in each graph correspond to correlations we would expect by chance after correcting for multiple comparisons. “significant” indicates the correlation value that would be statistically significant after Bonferonni corrections for multiple tests.

As expected, the open‐weight models performed worse than even the worst performing GPT‐4 method (OpenCLIP mean r(68) =  .37; Qwen‐3 mean r(68) =  .39; Qwen‐3 VL mean r(68) =  .50). Nevertheless, these correlations were statistically significant for 31, 35, and 40 out of 70 concepts for OpenCLIP, Qwen‐3, and Qwen‐3 VL, respectively, after applying a Bonferroni correction for multiple comparisons (Fig. 8). OpenCLIP and Qwen‐3 showed equivalent correlations with human ratings (t(69) = 0.64; p=  .52 for difference between mean correlations). Qwen‐3 VL, however, showed reliably higher positive correlations with the human data (mean r(68) =   .50) relative to the LLM variant, Qwen‐3 (mean r(68) =   .37; t(69) = 3.77, p<  .001). Qwen‐3 VL also generated associations that were more strongly correlated with human ratings than OpenCLIP (t(69) = 2.69; p<  .005). Since both Qwen‐3 variants are closely matched for confounding factors, such as number of parameters, model architecture, and language training corpora, this result suggests that the benefit arises from the joint influence of text and image information.

*Summary of Experiment 2B*. Experiment 2B showed that while smaller open‐weight models do not perform as well as GPT‐4 overall in predicting human color–concept associations, even simple nonautoregressive embedding models like OpenCLIP are capable of generating association ratings that are significantly correlated with those of humans. We further replicated a key analysis from Experiment 2A showing that multimodal variants of a given architecture, when prompted with both image and text, show better alignment than those that accept text‐only.

## 6 Experiment 3

As outlined in the outset of this paper, many prior studies have estimated human color–concept associations using information latent in images combined with various other natural‐language and machine‐learning techniques that require more than minimal supervision. To understand how such approaches compare to the results just described, we evaluated two state‐of‐the‐art methods described in the Introduction: the *image + category extrapolation* ^2^ approach described by Rathore et al. (2020) and the *colorization* approach introduced by Hu et al. (2023). Specifically, we were interested in whether (1) GPT‐4V would perform at par or better than bespoke models for estimating color–concept associations and (2) if GPT‐4V would perform notably better for abstract concepts that may not have reliable grounding in image databases.

### 6.1 Methods

Rathore et al. (2020) estimated color–concept association using information derived from the distribution of color pixels and their color category membership in images of concepts queried via Google Image search (expanding on previous methods from Lin et al., 2013). This study included few items overlapping with the current work, so we used the same approach applied to a new batch of Google images collected in 2025 to estimate associations for all 70 concepts. Further details can be found in Section E of the Supplementary Materials.

Hu et al. (2023) trained convolutional neural networks to colorize grayscale photos, and from these, estimated color–concept associations for 15 of the concepts and all 71 colors used in the current study. We compared how well the Hu et al. and the GPT‐4‐based estimates correlated with mean human judgments for these 15 items.

### 6.2 Results and discussion

Table 3 shows the correlation between estimated and true (i.e., human‐derived) color–concept associations generated from the best GPT‐4 approach (VLM with hex and image) versus each prior approach, tabulated on the same 15 concepts for each comparison (i.e., those overlapping with Hu et al.'s items).

**Table 3:** Correlations between human color–concept associations and estimated associations using the methods described in Hu et al. (2023), Rathore et al. (2020), and the current GPT‐4 study

Category	(Hu et al., 2023)	(Rathore et al., 2020)	GPT‐4V (Experiment 2)
Apple	0.69	0.42	**0.92**
Banana	0.88	0.87	**0.95**
Blueberry	0.85	0.84	**0.91**
Carrot	0.82	0.76	**0.83**
Celery	0.77	0.89	**0.94**
Cherry	0.62	0.78	**0.93**
Corn	0.81	0.80	**0.88**
Eggplant	0.49	0.05	**0.70**
Grape	0.12	0.17	**0.75**
Lemon	0.92	0.85	**0.94**
Mango	0.92	0.88	**0.94**
Mushroom	0.54	0.71	**0.84**
Peach	0.86	**0.89**	0.85
Strawberry	0.66	0.64	**0.78**
Watermelon	0.65	0.70	**0.96**

*Note*. Bold numbers correspond to the best correlation for that concept

In all cases, human–model estimates were reliably correlated, but estimates from GPT‐4V showed the highest correlation (mean r(69) =  .89; max r(69) =  .96; min r(69) =  .71), significantly better than both the image + category extrapolation approach (mean r(69) =  .75 (max r(69) =  .89; min r(69) =  .06; paired t(14) vs. GPT‐4V = 2.84, p <.05) and the colorization approach (mean r(69) =  .75; max r(69) =  .88; min r(69) =  .12; paired t(14) vs. GPT‐4V = 3.26, p <.01).

The 15 items in the preceding analysis are all concrete items, as it was previously thought that image‐based approaches would be ineffective in estimating color associations for abstract concepts (Lin et al., 2013). The image + category extrapolation method, however, relies on the results of queries to image search engines, which can return images for any search query regardless of concreteness. We, therefore, evaluated whether the image + category extrapolation approach reliably estimates human color associations for abstract concepts, and compared this approach to the GPT‐4V results from Experiment 2A.

**Fig. 9:**
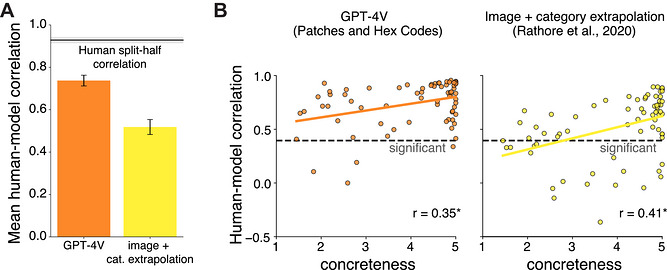
(A) Mean human–model association rating correlations for GPT‐4V using the patches and hex codes prompt method and Rathore et al.'s image + category extrapolation method. (B) Human–model association ratings correlation for each concept as a function of the concept's concreteness for GPT‐4V when prompted using color patches and hex codes and Rathore et al.'s model. “Significant” indicates the correlation value that would be statistically significant after Bonferonni corrections for multiple tests.

For the image + category extrapolation method, the mean correlation with human judgments across all concepts was r(69) =  .57, reliably weaker than the best performing GPT‐4V method (mean r(69) = .80; t(69) = 5.92, p <.001; Fig. 9A). Although this result shows that image‐based methods do not outperform GPT‐4 overall, it does not address whether this discrepancy arises from abstract concepts in particular. To address this question, we investigated the relationship between concreteness and human–model alignment for both GPT‐4V and the image + category extrapolation method (Fig. 9B). Both GPT‐4V and the image + category extrapolation method showed reliably higher human–model alignment for more concrete concepts (*r*(68) = .35 and *r*(68) = .41, *ps* < .05, respectively). To formally test whether Rathore et al.'s method yields worse alignment for abstract concepts than does GPT‐4V, we fit a linear regression model testing for an interaction between concreteness and model type when predicting human–model correlation across all 70 concepts. While we found significant main effects of concreteness (β = 0.10, *SE* = 0.02, t = 4.18, p <.001) and model type (β = 0.22, *SE* = 0.04, t = 5.45, p <.001), we failed to observe a significant interaction between these predictors (β = –0.04, *SE* = 0.04, t = –1.14, p =  .26). Thus, the lower predictive accuracy of image‐based approaches like image + category‐extrapolation cannot be attributed to poor performance on more abstract concepts per se—GPT‐4V shows better predictions regardless of concreteness.

*Summary of Experiment 3*. Experiment 3 showed that a multimodal language model, GPT‐4V, outperformed state‐of‐the‐art image‐based approaches to estimating color–concept associations. We also found that the weaker performance for Rathore et al.'s approach was not likely due to a particular inability to estimate associations for abstract concepts, compared with GPT‐4V.

## 7 Experiment 4

Thus far, our results show that color–concept associations generated by GPT‐4, while not reaching human split‐half reliability, nevertheless correlate strongly with human judgments, especially when the prompt includes images as well as hex codes for each color. In Experiment 4, we evaluated whether GPT‐4 estimates are *good enough* to predict human judgments in tasks that rely on color–concept associations. Specifically, we assessed whether GPT‐4V's estimates approximate human judgments sufficiently well to aid in the design of information visualizations (e.g., bar graphs) in ways that promote human ability to interpret color meaning.

**Fig. 10:**
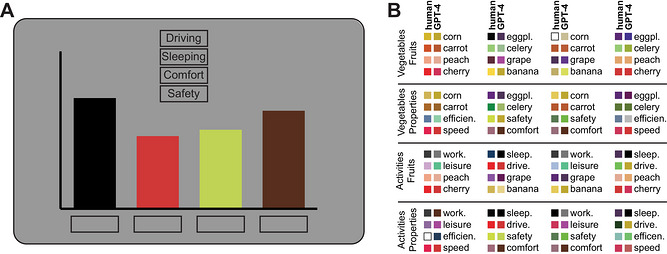
(A) Example trial in Experiment 4. Participants labeled each bar by clicking the label and dragging/dropping it in the box below the bar. (B) Concept quartets and corresponding palettes used to construct the stimuli for Experiment 4 (see text for details).

To this end, we presented participants with colored bar graphs representing different concepts and asked them to infer which color corresponds to each concept in the graph (Fig. 10A). Prior work found that observers could successfully infer which concepts corresponded to each color when the visualization was designed to optimize color–concept assignments using data from human color–concept associations (Mukherjee et al., 2022; Schloss et al., 2018; Schloss et al., 2021). The current experiment assessed whether humans perform comparably well at interpreting the color meanings when the color palettes for the visualization are instead optimized using GPT‐4V's color–concept associations (color patches and hex codes). If so, this would suggest that the color–concept association estimates from GPT‐4V are sufficiently human‐like to aid in the design of effective information visualizations for visual communication.

### 7.1 Methods

The experimental design and materials for this experiment were identical to those described in Mukherjee et al. (2022), which we describe below, except that the color palettes were generated by optimizing over GPT‐4V color–concept associations (using both hex and color‐patch prompts together) rather than human ratings (as used in Mukherjee et al.).

**Participants**. Ninety‐three participants (64 women, 24 men, 3 nonbinary, 2 other; 66 White, 11 Asian, 3 South Asian, 2 Native American/Hispanic, 1 Asian/Hispanic, 1 White/Hispanic, 1 White/Asian, 2 Hispanic, 1 Arab, 1 Jordanian, 4 unknown; M age = 18.44) from the University of Wisconsin‐Madison psychology subject pool participated in exchange for course credit. All gave informed consent, and the university IRB approved the protocol.

**Design, displays, and procedure**. The participants viewed bar graphs like that in Fig. 10 and were asked to match each of four concept words to the colored bar that best represented the word. On each trial, participants viewed a graph with four colored bars centered on the screen. The bars were 130px wide with randomly varied heights (between 260px and 300px) and placed 45px apart. Each bar had an unlabeled empty box under it. At the start of the trial, the four concept words were presented in 22px font and stacked vertically in random order above the graph. During the trial, the participants labeled each bar by clicking and dragging one of the concepts from the top and placing it in the box under the corresponding bar. They could also move concepts between bars and reset the trial if they felt they mislabeled a bar. Once they had labeled each bar, they could proceed to the next trial by pressing a button labeled “Next.”

Each participant completed 64 trials, which included 8 quartets × 8 color positions within each set based on a Latin square design. Trials were blocked and randomized within each block such that participants saw all quartets once in each block. At the end of the study, participants were asked if they had difficulty distinguishing between colors relative to the average person and if they considered themselves colorblind. All participants indicated normal color vision. The displays were generated using the Chart.js and jsPsych JavaScript libraries.

***Concept sets***. We used the 20 concepts studied by Mukherjee et al. (2022), a subset of the 70 concepts presented in the current work that included vegetables, fruits, activities, and properties (see Table 1). These categories are semantically disparate and span the full range of specificity and concreteness values from the larger set.

We created 16 concept quartets from this set of 20 by combining pairs of concepts from one category (e.g., vegetables) with pairs of concepts from another category (e.g., activities). These quartets could be grouped into four groups of Vegetables/Fruits, Vegetables/Properties, Activities/Fruits, and Activities/Properties. The set of all 16 quartets can be seen in Fig. 10B. The motivation behind this grouping of concepts was to create quartets that were mostly concrete, mostly abstract, or a mix of both. The first two quartets in each group (the first two columns in Fig. 10B) were presented to one group of participants (*n* = 43), while the remaining quartets (columns 3 and 4 in Fig. 10B) were shown to a second group (*n* = 50) to reduce the number of trials any one participant needed to complete.

***Color palettes***. We produced color palettes for each concept quartet using the same procedure as in Mukherjee et al. (2022) to make the colors as interpretable as possible (i.e., optimizing for “balanced merit” as defined in Schloss et al., 2018). However, unlike Mukherjee et al., who used human color–concept associations to optimize the color palettes, we used color–concept associations produced by GPT‐4V. For details on how we used color–concept associations to optimize palette design, see Mukherjee et al. (2022), summarized in Supplementary Materials Section F. Fig. 10B shows the optimal palettes generated from GPT‐4 ratings in the present study versus human ratings from Mukherjee et al. (2022).

***Catch trials***. To test for attention to the task, we also included a catch trial in each block, where the bars were colored a shade of red, yellow, green, and blue. The concept labels for these trials were “red,” “yellow,” “green,” and “blue,” making the matching task as simple as possible. We planned to exclude participants if their accuracy did not exceed 75%, but all participants performed at ceiling on these trials, so none were excluded.

### 7.2 Results and discussion

**Fig. 11:**
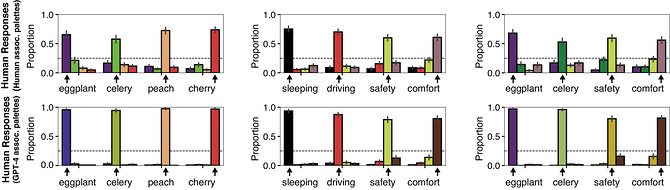
Proportion of times participants chose each color when palettes were generated using human association ratings (top row; adapted from Mukherjee et al., 2022) and GPT‐4V association ratings from Experiment 4 (bottom row, see Fig. G.1 in the Supplementary Materials for the full dataset). The correct response for each concept is marked by an arrow along the x‐axis. The colors of the bars correspond to the colors of the stimuli.

This experiment investigated whether people could interpret color palettes optimized using GPT‐4 color–concept associations as well as they can interpret color palettes that were optimized using human ratings. Fig. 11 (top) shows results reported in Mukherjee et al. (2022) (data collected in spring of 2020), in which participants interpreted color palettes generated from human ratings (this figure shows a representative set of quartets, the remaining data are shown in Fig. G.1). The plots for a given quartet show the proportion of trials that participants assigned each color to each concept in the set. The arrow below the x‐axis points up to the “correct” concept, which was the optimal color–concept pairing (see Supplementary Materials Section F). Fig. 11 (bottom) shows the corresponding data from the present experiment, in which participants judged color palettes optimized using data from GPT‐4V (see Fig. G.1 for the full dataset).

Overall, the participants were highly accurate at interpreting color palettes optimized using GPT‐4V color–concept associations (M = 0.81, SD = 0.20). In fact, the mean accuracy was higher than when previous participants interpreted color palettes optimized from human ratings (M = 0.66, SD = 0.15). To compare performance, we fit a mixed‐effect logistic regression model predicting assignment accuracy for each color–concept pair as a function of ratings source (human vs. GPT‐4; between‐subjects) with by‐subject random intercepts and by‐concept×palette random intercepts. The latter random effect allowed us to account for variability in accuracy for a given concept depending on the other concepts in the quartet. We found a significant effect of ratings source (β = 0.89, *SE* = 0.19, z = 4.66, p < .001), indicating that log odds of an accurate assignment was 0.89 points higher when the palette was generated from GPT‐4V ratings than from human ratings. Thus, not only did palettes optimized using GPT‐4V ratings support the interpretation of color meaning, but the present results also showed that GPT‐4V palettes outperformed palettes optimized using human ratings. We will return to potential explanations for this difference in the General Discussion.

*Summary of Experiment 4*. Taken together, Experiment 4 shows that color palettes optimized from GPT‐4V color–concept associations are highly interpretable by human observers, often more so than palettes generated from human ratings. This pattern held for concept sets that included concrete concepts, abstract concepts, or both. This finding is consistent with prior evidence that abstract concepts that lack observable colors can still be meaningfully represented using color, by virtue of their relative color–concept‐associations. The current results indicate that even if language models cannot perfectly capture human‐like color–concept associations, the estimates they do provide are *sufficiently human‐like* to generate color palettes for information visualizations that humans can easily interpret.

## 8 General discussion

Human beings have associations to varying degrees between essentially any concept and any color, a tendency that influences the meanings ascribed to colors in visual communication (Schloss et al., 2018; Schloss, 2024). To understand whether it is viable for a learning system to form color–concept associations from the visual and linguistic structure of the environment, we compared ratings generated by GPT‐4, a contemporary large‐language and vision‐language model, to averaged human ratings across 70 concepts spanning multiple concrete and abstract domains.

Across five prompting conditions, including conditions combining text and images, GPT‐4 produced color–concept association ratings that were remarkably well correlated with human judgments. This pattern was observed whether the model was probed solely with text (using color hex codes; Experiment 1) or with images (Experiment 2A, color patches), with the best performance observed when both formats were used together (Experiment 2A, color patches and hex codes). We also replicated the key advantage of using multimodal models for assessing color–concept associations using open‐weight variants of the models tested in Experiments 1 and 2A, further supporting the generalizability of our findings (Experiment 2B). Indeed, GPT‐4 reliably outperformed other state‐of‐the‐art methods for automatically estimating color–concept associations (Hu et al., 2023; Rathore et al., 2020) (Experiment 3). When used to select colors for data visualizations, the associations produced by GPT‐4 yielded palettes that human participants could easily interpret (Experiment 4). Model‐generated ratings showed lower correlations with human ratings for concepts that had lower color–concept specificity—that is, for concepts showing a relatively flat distribution of associations across colors. Intersubject agreement was also lowest for such concepts, so model‐generated ratings were similar to that of humans in that regard as well. Interestingly, the alignment between model‐ and human‐generated ratings did *not* vary with concept abstractness after controlling for the specificity effect, suggesting that GPT‐4 approximates human color associations equally well for both concrete and abstract items. Thus, with regard to the pragmatic aims of this work, the results indicate that GPT‐4‐derived color–concept associations approximate those of humans (a) better than other contemporary methods and (b) sufficiently well to aid in the design of effective visualizations (when used as input into models of assignment inference for interpreting color meaning in visualizations; see Schloss et al., 2018). Thus, large VLMs may serve as an easy, effective, accessible, and comparatively affordable tool for designing interpretable colors for visual communication.

With regard to the more theoretical aim of the work, we have noted several ways in which model‐generated ratings are similar to human ratings: (1) these models can rate association strengths for essentially any color and any concept; (2) the ratings generated correlate significantly and relatively highly with mean ratings produced by humans; (3) model agreement with human ratings is higher for high‐specificity concepts, just as intersubject agreement is higher for high‐specificity concepts; and (4) visualizations designed on the basis of model‐generated associations are easily interpretable to humans. At the same time, model‐generated ratings, even when high, never matched the degree of intersubject agreement, and for some concepts, fell fairly far short of this ceiling. What implications do these findings have for our understanding of human color–concept association, if any?

First, the work establishes that naturally arising web‐scale information environments contain sufficient information for a learning system to acquire color–concept associations that approximate those of humans. The demonstration is important because it illustrates that even highly abstract kinds of implicit knowledge can be learned from the visual and linguistic structure of the environment. Models of conceptual knowledge acquisition have long assumed that people acquire concrete object concepts by learning patterns of association among their various perceived or described attributes (Rosch, 1975; Rogers & McClelland, 2004; Rogers, 2024), and recent work has shown that such learning can also support color–concept associations for concrete objects (Schoenlein & Schloss, 2022). Others have hypothesized that broader learning of higher‐order environmental structure might likewise support highly abstract forms of knowledge (Liu et al., 2025), but it has not previously been clear how this hypothesis might be tested. The current results show that one variety of abstract knowledge—associations between colors and concepts such as “night,” “love,” and “driving”—is latent in the structure of web‐scale language and vision environments.

Second, the results provide an existence proof that one form of learning system, namely, a vision‐language transformer, can learn and exploit such structure without initial constraints explicitly designed to promote such learning. Of course, any learning system brings with it initial constraints that determine what structure it discovers in data; however, whatever the constraints imposed by GPT‐4, they certainly were not selected specifically to allow the model to capture human color–concept association patterns. The close approximation of human color–concept associations instead must have emerged as a side‐effect of the model architecture, structure in the data, and the particular objectives on which the model was trained—that is, next‐token prediction and reinforcement learning from human feedback —with the goal of generating human‐like patterns of conversational interactions. Moreover, the color–concept association patterns so acquired align better with human‐perceived associations than did those of systems that *are* explicitly designed to learn such associations from data. Together, these observations establish the face validity of one hypothesis about human color–concept associations: that these arise as a byproduct of learning high‐order correlational structure across vision and language.

Finally, our results suggest that such abstraction depends upon cross‐modal learning that integrates language and vision. While the text‐only models explored in Experiment 1 generated ratings highly correlated with human judgments for most concepts, they were probed using hex codes that designate highly specific colors. Such codes are not a part of ordinary natural language, but are used by visual rendering formalisms such as scalable vector graphics, providing a kind of text analog to vision in LLMs. Visual rendering languages self‐evidently do not encompass most natural language concepts (like “night,” “love” etc.)—thus, the ability to generate accurate color–concept ratings for colors designated in a hex code and concepts designated with natural language terms must arise from integrated learning about both (Du et al., 2025). Additionally, alignment with human judgments improved significantly when the model was prompted with both hex codes and bitmap images, further demonstrating that integration of linguistic, text‐based information with visual information produces more human‐like behavior. In this sense, the current work is consistent with models proposing that abstract conceptual representations arise from cross‐modal learning about the structure of the environment (Du et al., 2025; Ralph, Jefferies, Patterson, & Rogers, 2017; Patterson, Nestor, & Rogers, 2007; Rogers et al., 2004; Xu et al., 2025).

*Limitations and future directions*. What are we to make of the fact that human‐to‐human agreement is systematically higher than model‐to‐human agreement? To some extent, such a discrepancy is unsurprising, since there are many aspects of shared human experience not reflected in web‐scale text and image corpora: sound, motion, affect, and action, among others. Previous work has also pointed to the lack of rich modal training data (e.g., olfactory, motor, gustatory) as one reason why modern multimodal AI systems are not fully aligned with humans, specifically on dimensions that require sensorimotor grounding (Xu et al., 2025). Even within the domain of vision, investigations of the dimensions underlying VLMs’ conceptual structure often fail to reveal salient dimensions relating to color (Du et al., 2025). Coupled with failures on other simple visual tasks (Ramachandran et al., 2025; Rahmanzadehgervi, Bolton, Taesiri, & Nguyen, 2024), these results indicate that this class of models does not have perfect coverage over vision as a domain either. To the extent that fine‐grained color–concept associations—that is, ratings between concepts and specific colors—require some degree of grounding in visual experience, we might expect current models to fall short. Such discrepancies may diminish as models advance to better capture these aspects of human experience; it is also possible, of course, that learning of environmental structure gets a system much of the way there, but cannot completely explain all aspects of human color–concept association.

While GPT‐4 and GPT‐4V were able to provide valid responses within the scale ranges in the prompted instructions for the majority of trials, in one case, the model provided the exact same rating for all colors (the concept “beside” when evaluated using GPT‐4V). This failure to use the full range of the scale departs from behavior observed in humans and the text‐only GPT‐4 model. Thus, there might still be some concepts where the current zero‐shot method fails. Since our focus was on evaluating each model in a highly controlled manner, we did not seek to optimize the prompt for specific failure modes; alignment for such edge cases may be improved using prompt engineering methods (Khattab et al., 2023).

Finally, we note that, while the models we have evaluated can approximate human color–concept associations quite well, this does not imply that the processes by which the models generate such ratings are good analogs of the psychological processes that give rise to human ratings. Certainly contemporary LLM/VLMs and humans differ in many ways in how they learn, represent, and process information.

Nevertheless, our results demonstrate that the associations generated by GPT‐4V are sufficient to guide selection of effective colors for visual communication—indeed, they open the possibility that palettes optimized based on model color–concept associations may be more effective in this regard than those based on human judgments. Specifically, participants showed higher accuracy at mapping concepts to colored bars in bar charts when palettes were optimized based on GPT‐4V's color–concept associations, compared to a prior study where palettes were optimized using human associations (Mukherjee et al., 2022). We note, however, since the prior study was conducted online during the COVID pandemic, when workers were shown to be less attentive (Arechar & Rand, 2021), there is the possibility that accuracies were slightly deflated. Testing the possibility that palettes based on model associations are more effective than those based on human associations will require comparison on more comparable populations.

The current results comparing human and model color–concept associations speak to a specific human population, namely, university students from the United States who are fluent in English. Our findings might not generalize to other languages or to cultures that have distinct color–concept associations from those observed in our sampled population. Conversely, it may be that associations generated by LLMs/VLMs are *more* representative of a broader population than are those estimated from human participants on crowd‐sourcing platforms like Amazon Mechanical Turk. The web‐scale data used to train contemporary models represent a far vaster and more diverse variety of human behavior than the particular samples of participants recruited from university subject pools or online crowd‐sourcing platforms. Future work should assess how color–concept associations vary across languages and cultures (Adams & Osgood, 1973; Jonauskaite & Mohr, 2025; Palmer et al., 2013; Soriano & Valenzuela, 2009; Tham et al., 2020, Ou et al., 2004), and whether language‐vision models are more representative of, or can better capture, such variability.

The rapid advancement of multilingual VLMs (Chen et al., 2022) opens the door to such research, and raises the possibility of generating culture‐ or language‐specific recommendations for use of color in data visualizations. We further note that the main models we have focused on, GPT‐4 and GPT‐4V, are closed and proprietary. Consequently, it remains unclear precisely what aspects of the environmental structure or learning system are necessary or sufficient to support this capability. The open‐weights models we have considered—Qwen‐3, Qwen‐3 VL, and OpenCLIP—while less opaque than GPT‐4, were also less performant likely due to their smaller size. Nevertheless, all three models generated color–concept associations that were significantly correlated with human ratings for many concepts; and the contrast of Qwen‐3 versus Qwen‐3 VL showed improved performance for prompts combining text‐ and vision‐based indicators of color — suggesting that key observations from GPT‐4 are replicable in open models. A systematic survey of all frontier models such as Claude, Gemini, and others, as well as larger open‐weight models, might further aid in understanding how general this capability is across model variants; however, progress in understanding how environmental structure, architecture, and learning algorithm combine to support abstract knowledge acquisition will require more controlled studies and comparisons of open‐weight models. At present, we view our results as uncovering a competence within a single family of both frontier and open‐weight language‐vision models.

Moreover, our results are important to the field of information visualization, which has shown that optimizing color palettes for interpretability requires having color–concept association data sampled broadly over color space. That is, estimating only the top most associated colors is insufficient, given that sometimes the optimal color palettes are those that assign individual concepts to weakly associated colors (see Schloss et al., 2018). Collecting such color–concept association data from humans is highly labor‐intensive, but our results show that LLMs/VLMs can automate this process effectively. Moreover, the methods we have developed here can be easily extended to new models as they arise. Should such models show even better alignment with human color–concept associations, the resulting estimated ratings can, in principle, be immediately deployed for palette design (Mukherjee et al., 2022; Schloss et al., 2018). Finally, while we explored a variety of prompting methods spanning the text and image modality, there remains a vast universe of optimizations that could be performed to further improve alignment to human color–concept associations, including prompt‐tuning, in‐context learning (Brown et al., 2020), or lightweight and efficient fine‐tuning strategies such as LoRA (Hu et al., 2021) on open‐sourced models.

## 9 Conclusion

We began this paper by asking whether high‐order structure exists in real‐world language and perceptual experiences that can enable a learning system to produce color–concept association that approximate those of humans. We have shown that such associations are latent in the statistical structure of natural language and color information present in web‐scale corpora, and that large‐language/language‐vision models like GPT‐4, which learn from high‐order structure in such corpora, can produce color–concept association ratings that correlate strongly with human ratings. From a pragmatic perspective, contemporary AI can provide a useful tool for automatically estimating color–concept associations across a broad range of concepts, which can be used to predict aspects of visual cognition that rely on such associations (e.g., object recognition, color preferences, interpretation of information visualizations). From a theoretical perspective, the work establishes the face validity of the hypothesis that color–concept associations can be acquired through learning high‐order structure across language and vision. To empirically test this hypothesis, future human behavioral experiments will need to carefully manipulate the structure and frequency of color–concept co‐occurrences and test the effect of varied statistical co‐occurrences on learned associations. In the meantime, the use of contemporary VLMs can provide a useful tool for designing optimal palettes to support visual communication.

## Data and code availability statement

Data and code will be made available in the following Github Repository ‐ https://github.com/SchlossVRL/gpt‐cca.

## Author contributions

K.M., T.T.R., and K.B.S. formulated ideas and designed the human and model experiments, and planned analyses, and interpreted the results. K.M. and A.M. conducted the experiments and analyzed the data. All authors contributed in writing the paper.

## Conflicts of interest

We declare no conflicts of interest.

## Ethics approval statement

All studies reported were conducted in accordance with the University of Wisconsin‐Madison IRB.

## Supporting information


Supporting Information

## Notes

1 All code and materials for experiments reported in this paper can be found at— https://github.com/SchlossVRL/gpt‐cca.

2 We call this approach “image + category extrapolation” as shorthand, as it uses both sector‐shaped tolerances around the pixel color input and color category membership of the pixel colors (see Supplementary Materials Section E for details).
