# Supplementary material for: Large Language Models Estimate Fine‐Grained Human Color–Concept Associations
Source: Cogn Sci. 2026 Jun 22;50(6):e70219. doi: 10.1111/cogs.70219 (PMC13286014; doi:10.1111/cogs.70219)
Supplement: Supplementary file 1 — Supporting Information [file COGS-50-e70219-s001.pdf]

SUPPLEMENTARY MATERIALS

A Distributions of specificity and concreteness values for concepts used.

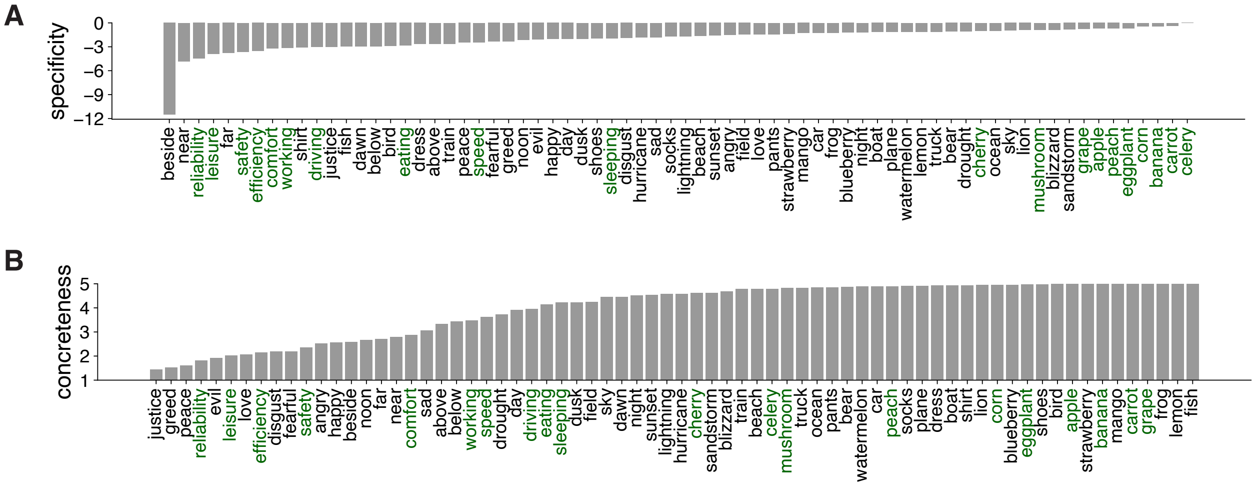

Figure A.1: (A) Distribution of specificity values for all 70 concepts. (B) Distribution of concreteness values for all 70 concepts. Concepts highlighted in green text indicate the 20 concepts used in Mukherjee et al. (2021) [1] and Experiment 4 in the present work. The subset of 20 concepts spanned both concreteness and specificity values.

B Effect of multiple ratings on human-model alignment

Given that specificity helped explain the degree to which human and GPT-4 ratings were aligned, we investigated whether specificity could also explain why some concepts benefited from multiple ratings more than others. To test this, we computed the mean correlation for each concept as a function of the number of ratings used to compute the correlation (0 to 10; Figure B.1). The curves show large concept-to-concept variance in terms of correlation with human ratings, which is predicted by the concept's specificity. While some concepts show higher correlations as a function of number of ratings, these gains appear to be limited with most correlations stabilizing following about three ratings and with many low-specificity concepts plateauing quite early. These results suggest that while acquiring multiple ratings improves performance for estimating color-concept associations when using text-based representations of color, the margin of improvement is narrow (mean r = 0.69 for the multiple ratings condition vs. mean r = 0.67 for the no anchoring condition, the next best performing method) and does not always benefit low-specificity concepts.

C Measuring differences between model and human ratings in terms of root mean square errors

While we focus on correlations between human and model ratings as the key measure of interest, due to the *relative* differences between color associations between concepts being a key driver of the meanings people ascribe to colors [2, 3, 1, 4], here we also report the root mean squared errors (RMSE) between mean human ratings and model ratings. RMSE captures how well the exact ratings generated by a given model for a given color concept pair diverge from the human 'ground truth' and we computed this metric for each concept individually and report the mean RMSE across concepts in Figure C.1. Generally, we found concordance between these results and those we report in the main text using Pearson correlations. Notable differences included the observation the Qwen models did not show any differences in RMSE ($t(69)$ = 0.88, p = 0.38) despite the VLM variant (Qwen-VL) showing significantly higher correlations than the language-only model. The text-only GPT-4 model with multiple ratings also showed the lowest RMSE, even lower than GPT-4V when both hex codes and color patch images were provided ($t(69)$ = 2.29, p <0.05) departing from the

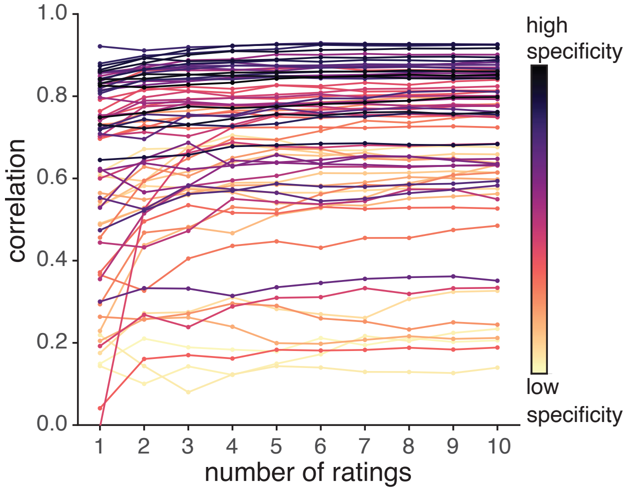

Figure B.1: Average correlations between human color-concept association ratings and predicted GPT-4 ratings across all concepts as a function of the number of ratings acquired in Experiment 3. Each line corresponds to a different concept and the color of the line corresponds to the specificity of the concept.

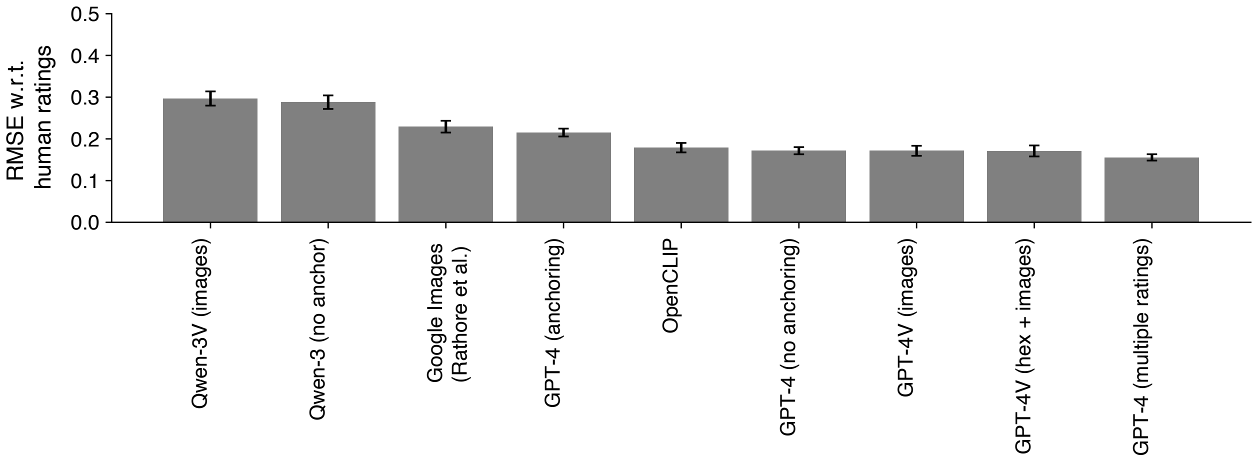

Figure C.1: Mean RMSE values between model color-concept association ratings and human ratings. Models are sorted in descending order and errorbars correspond to 95% confidence intervals.

observation that GPT-4V prompted with hexcodes and color patches achieved the highest correlations. This shows that with the current prompting regimes, correlation-based and error-based metrics are not always in perfect agreement.

D Effect of different prompt templates for OpenCLIP evaluations

In the main text, we use just the concept word as the text prompt provided to OpenCLIP ViT-B-32 when computing similarities between text embeddings and color patch image embeddings. This was done because this set up most closely mirrors the trials participants completed where they were only shown a color patch, a concept word, and were asked to provide a rating. However, it remains possible that other prompts may have led to significanlty better alignment with human ratings. To test this hypothesis, we repeated the experiment, with two alternative text prompt templates. The templates were as follows -

```
Alternaive Template 1:  "When I think of the colors of {concept}, I think of ..."
```

and

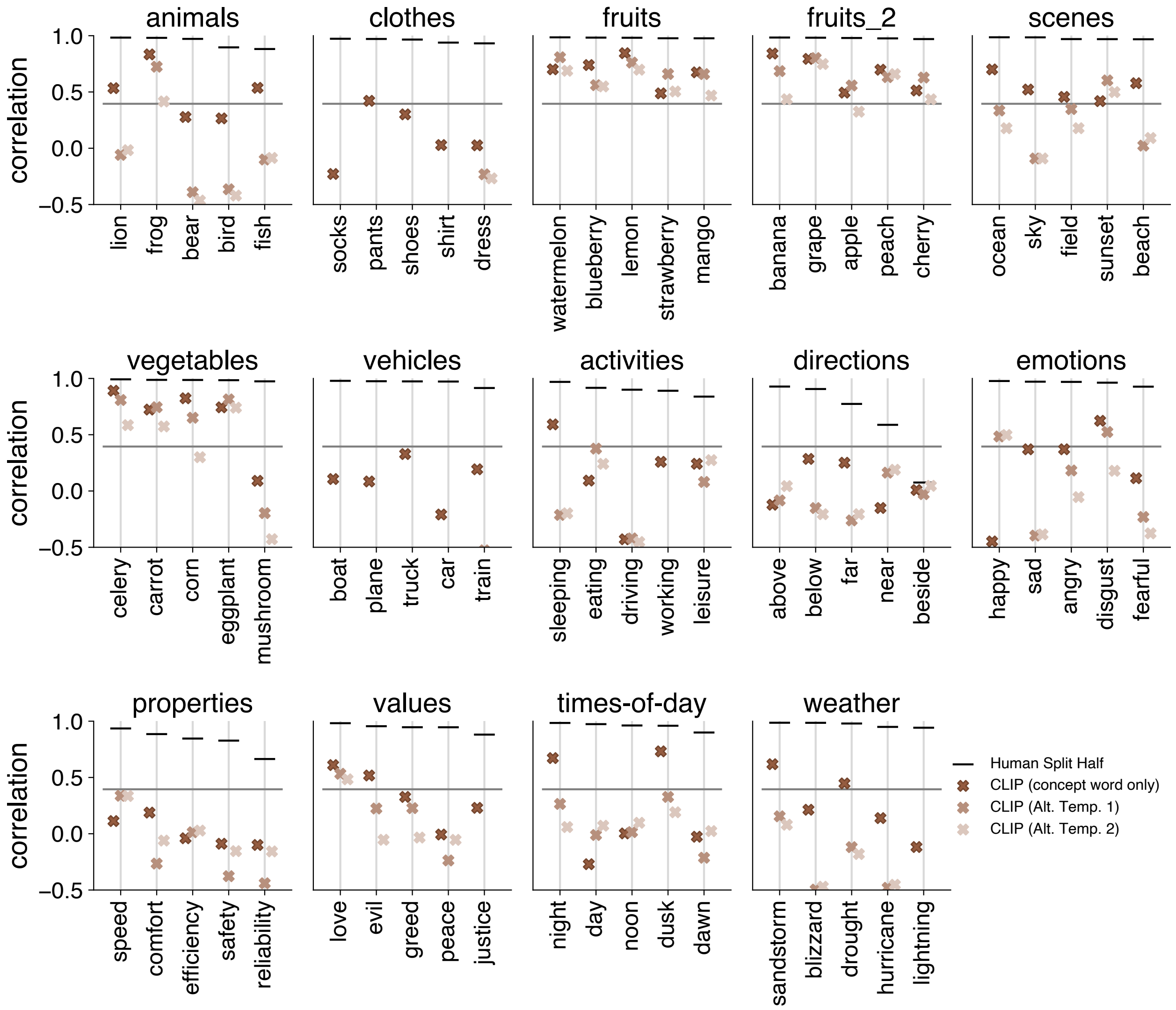

Figure D.1: Correlations between average human color-concept association ratings and predicted ratings from OpenCLIP under different prompting templates. Refer to the text for the wording of the prompts. Black lines above each concept correspond to the average human split-half reliability for that concept. Gray lines in each graph correspond to correlations we would expect by chance after correcting for multiple comparisons.

```
Alternaive Template 2: "The color that is associated with {concept} is ..."
```

, where `concept` would be replaced with the concept whose associations were being measured. The pattern of correlations with human ratings can be seen in Figure D.1. As can be seen, the overall pattern of correlations was much lower when using these alternative prompts (mean $rs(69)$ = 0.04 and -0.04 respectively). While this does not preclude the possibility of there being an optimal prompt that would lead to better alignment, these results show that providing just the concept names as input does not result in systematically worse performance than more verbose, targeted prompts eliciting color associations.

E Estimating color-concept associations using Google Images (Rathore et al., 2020) [5]

Here we provide a brief description of the Rathore et al.'s method for estimating color-concept associations using image databases. Refer to the original paper for a more detailed explanation.

Given a concept, the first step is to download n images of the concept from an image database such as Google Images. The authors found that there was little improvement in performance beyond using n = 10 images. The next step is to

define the set of colors for which associations are to be estimated. In the original work Rathore et al. use the `UW-58` a subset of the `UW-71` used in the current work. Next, several candidate image statistics-based features relevant for estimating color concept association are derived from the concept images. These features help define which pixels in the image should 'count' towards the association between the concept and a given color in the color set. One class of features referred to as 'ball' features counted a pixel towards a particular color if the color of the pixel fell within a radius δr of the target color in CIELAB space. Another class of features were based on cylindrical sectors around the target color as opposed to Cartesian balls. This was due to the fact the dimensions of perceptual color space are cylindrical with hue defining the angle, chroma the radius, and lightness the height of the slice of color space being viewed. A pixel in a given image counted towards the association between the concept and a target color if the pixel's color fell within the sector for the target color. The last class of features were based on carving color space into 11 categories based on the method described by [6]. If the pixel's color belonged to the same color category as the target color, then it counted towards its association. The authors generated several candidate features by varying the width and coverage of the ball and sector features and varying what parts of the images were under consideration when estimating associations (5 conditions using between 20% - 100% of the total image measured from the center and one condition using a segmentation algorithm to extract figure from the image relative to a background). Using cross-validated sparse regression, they found the optimal set of 4 features which included two sector and one color category features plus a constant offset. They further used linear regression models to estimate the optimal weights on these features for predicting human color-concept association ratings. We used this variant of the model – that used two sector, color category, and a constant as features – for Experiment 3 in the present work. After image statistics were extracted from the Google Images, we supplied them as input into the model (with trained weights), which in turn generated estimated associations with each of the specified colors in the `UW-71` color library.

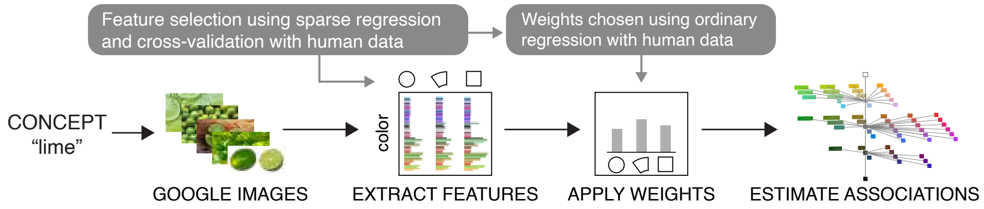

Figure E.1: Rathore et al.'s procedure for estimating color-concept association from Google Images. Adapted from [5].

F Designing optimal palettes based on color-concept associations

We generated the optimal palette for each set of concepts by solving an assignment problem [7] that maximized the "merit" (or goodness) between each concept and each color from a color library (i.e., the concepts in a quartet and the colors in the UW-71 color library). In assignment problems, every possible pairing of items in one category (e.g., colors) i and another category (e.g., concepts) j is given a numerical *merit score* m_{ij}. In our case, larger merit scores correspond to better 'goodness' between a color-concept mapping. Solving an assignment problem means finding the pairing of items that, in this case, maximizes the sum of the merit scores of all chosen pairs [8, 7, 9].

Although there are many possible ways to define merit, Schloss et al. [2](and later [1]) found that a *balanced merit function* was an effective for optimizing palettes for human interpretability. The function was defined as follows -

$$m_{ij} := a_{ij} - \max_{k \neq j} a_{ik}. \tag{1}$$

The balanced merit score for a given color-concept pair is thus the association strength for that pair, minus the association strength between that color and the next most strongly associated concept. In order for m_{ij} to be large, color i should be strongly associated with concept j and weakly associated with all other concepts.

Schloss et al. [2] found that palettes designed using balanced merit were easier to interpret than palettes designed using merit based on associations in isolation (*isolated merit function*).

In the present study, for each concept quartet, we used color-concept associations from GPT-4V to compute balanced merit for each possible color-concept pairing. We then solved an assignment problem over all 71 colors in the UW-71 color library to obtain the optimal color palette for each concept quartet.

G Human responses to assignment inference task for palettes generated from human and GPT-4V association ratings

Figure G.1 shows patterns of human responses for the bar graph interpretation task conducted in Experiment 4 for all concept quartets. Results are shown for palettes generated from human (left columns) and GPT-4V (right columns) association ratings.

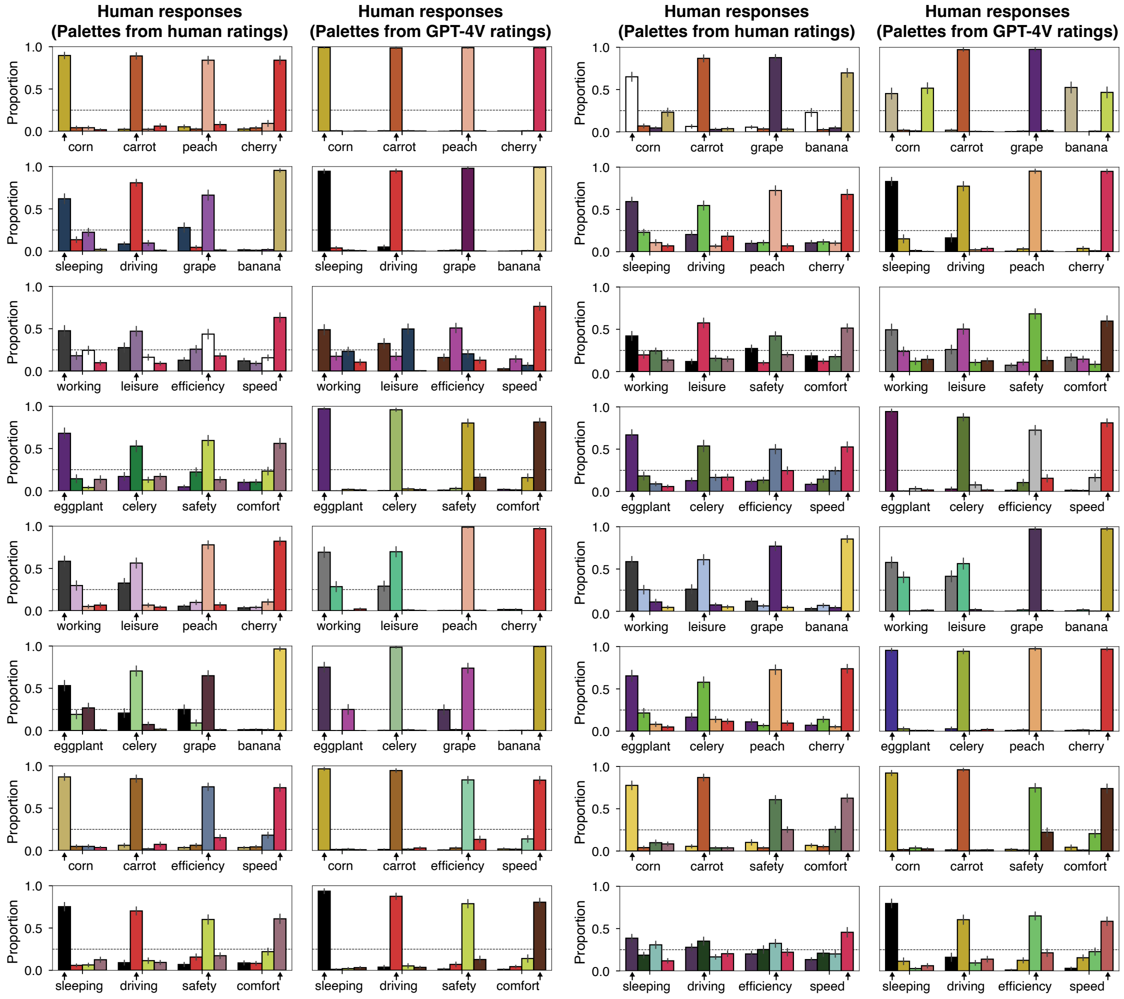

Figure G.1: Participant responses to palettes generated from human association ratings (left; data from Mukherjee et al. (2021)) and palettes generating from GPT-4V association ratings (Experiment 2b). Each pair of bar plots corresponds to one of the color-concept sets from Experiment 4. The solid black line across each graph corresponds to making an assignment by chance (.25). The correct response color for each concept is marked along the x-axis by an arrow. Error bars represent the ± standard errors of the mean accuracies.

H Does human performance on GPT-4 palettes exhibit a relationship between semantic discriminability [3] and accuracy?

Previous work found that color palettes that were more *semantically discriminable* were also ones where people were more likely to accurately decode the encoded mapping. Semantic discriminability refers to the ability to discriminate colors in terms of their meaning [3]. One way this has been operationalized in prior work is to measure how much better one mapping between colors and concepts is relative to the other (in the case of 2 concepts and 2 colors), regardless of what the 'correct' mapping intended by a designer is. This notion was later generalized to n concepts and n colors[1].

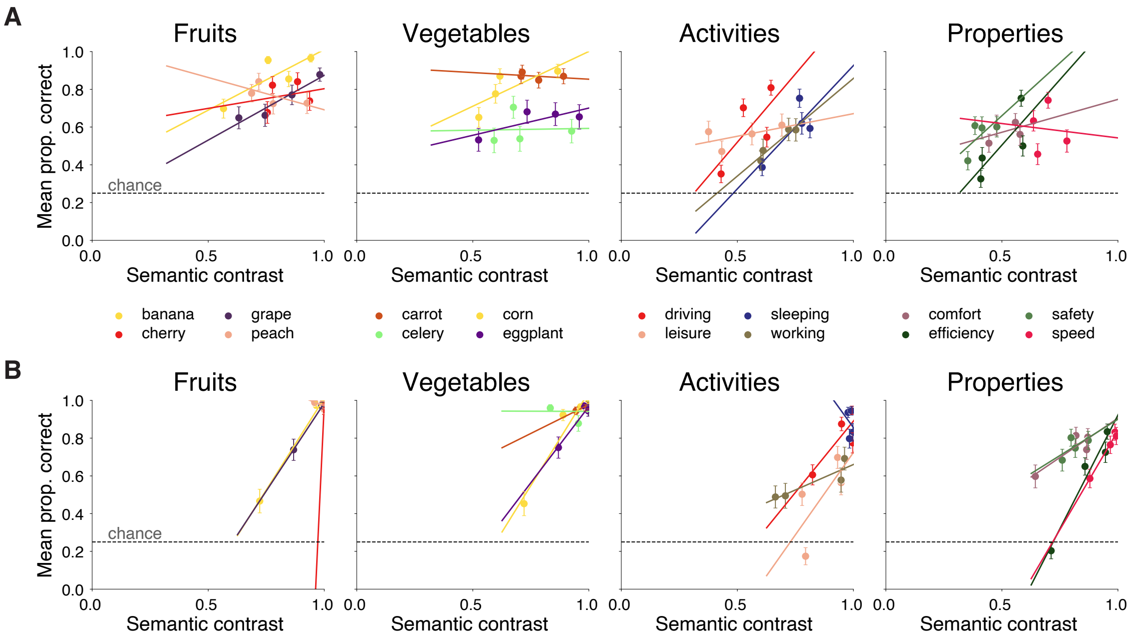

Figure H.1: (B) Mean proportion correct (accuracy) for each concept in each set as a function of semantic contrast of its target color (lines correspond to best fit lines for each concept) when human ratings are used for palette generation. Points for a given concept and corresponding best fit line are shown in the same color for ease of visual grouping (these colors were not necessarily the colors shown in the experiment). Figure adapted from Mukherjee et al. 2021 [1]). (C) Mean proportion correct for each concept in each set as a function of the semantic contrast of its target color when GPT-4V ratings are used for palette generation. Gray horizontal lines correspond to chance (.25) and error bars represent ± standard errors of the means.

Mukherjee et al. (2021) [1] refer to one such measure of a given colors' semantic discriminability among a set of n colors as the *semantic contrast* of that color and it is computed as follows for a given concept set in the data we present:

- First we compute the optimal assignment between the concept set and `UW-71` colors using the mean GPT-4V association rating from Experiment 2.
- Next, we sample association ratings from the mean association ratings matrix. We assume that the values in cell of the matrix is normally distributed with the mean equal to the observed mean ($x_{i,j}$ in that cell and variance equal to $1.4 \times x_{i,j} \times (1 - x_{i,j})$ [1]. Using this sampled matrix, we once again solve the assignment problem and record its solutions
- Finally we repeat step 2 a large number of times (10,000) and count the proportion of times each color was assigned to the same concept as in the optimal assignment. This proportion is a measure of each color's semantic contrast within a given concept set and ranges from 0 to 1.

Overall, we found that the patterns of responses generated by our model of assignment inference were strongly correlated with the human response data ($r(255) = 0.96$, $p < 0.001$) showing that our model was a good fit to human behavior. Critically, we also found that the greater the semantic contrast of a color in a palette the higher the average accuracy for mapping that color to the correct concept. This is exemplified in Figure H.1A, which shows the relationship between semantic contrast and assignment accuracy for human ratings-based palettes from Mukherjee et al. (2021). The slopes for most concepts were positive and they had indicating that colors that were more semantically discriminable were more often matched to the correct concept. Further, they had shown that both the specificity of the target concept and the semantic contrast were predictive of accuracy. We sought to replicate this analysis. Figure H.1B shows the same semantic contrast versus accuracy plot but for GPT-4 ratings-based palettes. Here, we again find that nearly all best-fit lines are positive replicating the results from human-ratings based palettes.

We analyzed the pattern of accuracies in finer granularity using a mixed-effect logistic regression model modeling the probability of correctly assigning a concept to the optimal color in a given concept set using three factors — semantic

[1]This has been previously shown to be a good fit for experimental data of this kind [3, 1]

**Fixed Effects**	β	SE	z	p
Intercept	2.21	0.14	15.85	< 0.001
Specificity	0.05	0.09	0.53	0.60
Semantic Contrast	1.02	0.10	9.90	< 0.001
Association Strength	0.37	0.10	3.63	<0.001

Table H.1: Results from logistic mixed-effect model predicting accuracy from specificity of the concept, semantic contrast of the concept's correct color, and association between the concept and its target color.

contrast of the optimal for that concept, specificity of the concept, and association strength between the concept and its correct color from Experiment 3 (previously shown to influence accuracy in similar tasks). We also included by-subject random intercepts and by-subject random slopes for each factor and z-scored the individual predictors to put them on the same scale. It should be noted that here specificity was computed with respect to the GPT-4V association ratings from Experiment 3. As shown in Table H.1, the effects of semantic contrast and association strength were significant, while the effect of specificity was not. This result differs from the findings reported by Mukherjee et al. (2021) [1], where no significant effect of association strength was observed, and a significant effect of specificity was reported. However, the effect of semantic contrast was the strongest in both studies highlighting the importance of context in determining how interpretable a given encoding system is.

I Modeling color concept associations using color space regression models

In this paper our goal was to test whether LLMs like GPT-4 can produce human-like color-concept association ratings. In this context, it is helpful to characterize human color-concept associations. One way to do this is to compute metrics on the distribution of associations for a given concept such as *specificity*. Another way, that provides detailed information about a concept's associations in terms of how they relate to color space properties is to use color space (colorimetric) regression models that use coordinates in color space to explain patterns of data sampled across color space. These models were first developed and tested to model human color preferences [10, 11], but have recently been extended to model human color-concept association ratings (refer to supplementary materials in Schoenlein et al. (2022) [4] for more details). Schloss et al. [12] showed that a cylindrical model in CIELCh space with two harmonics best explained color preference data and this same model was subsequently used by Schoenlein et al. (2022) [4] to model people's color-concept associations. The model consists of seven predictors — lightness (L), chroma (C), the first harmonic of hue angle (sin(h) and cos(h)), the second harmonic of hue angle (sin(2h) and cos(2h)), and a constant (k). These models can be use to effectively estimate human color-concept associations (measured via model fit) and investigating the coefficients or weights of the models highlight the relationship between color space properties and association ratings on a concept-by-concept basis. Separate models were fit for each concept where the predictors were the 6 colorimetric properties of each of the `UW-71` colors plus the constant and the predicted value was the association rating between the concept and that color. Models were fit using ordinary least squares (OLS). The quality of the model fits were assessed by computing the correlation between true ratings and predicted ratings using the fitted coefficients. Figure I.1 shows estimated associations and visual depictions of model weights for four models fit to four difference concepts. Refer to table I.1 for coefficients for all concepts.

**Concept**	**L**	**C**	**cos(H)**	**sin(H)**	**cos(2H)**	**sin(2H)**	**k**
above	0.005	0.001	-0.051	-0.104	-0.104	-0.104	0.183
angry	-0.005	0.001	0.063	0.099	0.099	0.099	0.526
apple	-0.001	0.003	-0.026	0.201	0.201	0.201	0.151
banana	0.002	-0.0	0.027	0.177	0.177	0.177	0.062
beach	0.004	-0.001	-0.106	-0.146	-0.146	-0.146	0.292
bear	-0.003	-0.004	0.048	0.167	0.167	0.167	0.656
below	-0.005	-0.002	-0.021	0.039	0.039	0.039	0.808
beside	0.001	-0.0	-0.007	-0.027	-0.027	-0.027	0.431
bird	0.0	-0.002	0.003	0.023	0.023	0.023	0.553

blizzard	0.003	-0.004	-0.062	-0.272	-0.272	-0.272	0.422
blueberry	-0.001	-0.0	-0.104	-0.232	-0.232	-0.232	0.448
boat	0.001	-0.004	-0.038	-0.159	-0.159	-0.159	0.513
car	-0.0	-0.003	0.05	-0.009	-0.009	-0.009	0.485
carrot	-0.001	0.002	-0.026	0.209	0.209	0.209	0.22
celery	-0.0	0.0	-0.155	0.19	0.19	0.19	0.182
cherry	-0.002	0.003	0.057	0.093	0.093	0.093	0.179
comfort	0.003	-0.002	0.022	-0.07	-0.07	-0.07	0.43
corn	0.001	-0.0	0.0	0.206	0.206	0.206	0.103
dawn	0.003	-0.001	0.054	-0.033	-0.033	-0.033	0.332
day	0.007	0.001	-0.068	-0.083	-0.083	-0.083	0.087
disgust	-0.004	-0.001	-0.04	0.201	0.201	0.201	0.652
dress	0.002	-0.001	0.077	-0.112	-0.112	-0.112	0.393
driving	-0.0	-0.0	-0.041	0.041	0.041	0.041	0.475
drought	-0.0	-0.003	0.11	0.193	0.193	0.193	0.469
dusk	-0.005	-0.002	0.039	-0.051	-0.051	-0.051	0.815
eating	0.0	0.001	-0.005	0.102	0.102	0.102	0.344
efficiency	0.003	0.001	-0.033	-0.055	-0.055	-0.055	0.266
eggplant	-0.002	0.0	0.062	0.019	0.019	0.019	0.332
evil	-0.006	-0.0	0.006	0.084	0.084	0.084	0.699
far	-0.002	-0.002	-0.013	-0.014	-0.014	-0.014	0.676
fearful	-0.004	-0.001	0.022	0.046	0.046	0.046	0.63
field	-0.001	-0.0	-0.079	0.175	0.175	0.175	0.406
fish	-0.0	-0.001	-0.087	-0.071	-0.071	-0.071	0.548
frog	-0.003	-0.001	-0.179	0.166	0.166	0.166	0.547
grape	-0.002	0.002	0.034	0.03	0.03	0.03	0.268
greed	-0.004	-0.0	-0.05	0.133	0.133	0.133	0.633
happy	0.006	0.004	-0.006	-0.091	-0.091	-0.091	-0.015
hurricane	-0.002	-0.002	-0.118	-0.128	-0.128	-0.128	0.675
justice	0.001	-0.002	0.049	-0.056	-0.056	-0.056	0.441
leisure	0.002	-0.0	-0.045	-0.092	-0.092	-0.092	0.42
lemon	0.003	-0.0	0.036	0.115	0.115	0.115	0.051
lightning	0.004	-0.002	0.048	-0.054	-0.054	-0.054	0.202
lion	-0.0	-0.001	0.102	0.212	0.212	0.212	0.389
love	0.003	0.003	0.167	-0.075	-0.075	-0.075	0.069
mango	0.001	0.002	0.029	0.164	0.164	0.164	0.132
mushroom	0.0	-0.004	0.104	0.17	0.17	0.17	0.387
near	0.002	0.001	0.007	-0.025	-0.025	-0.025	0.304
night	-0.007	-0.003	-0.013	-0.053	-0.053	-0.053	0.923
noon	0.006	0.001	-0.037	-0.056	-0.056	-0.056	0.1

ocean	0.0	-0.001	-0.247	-0.29	-0.29	-0.29	0.514
pants	-0.001	-0.005	0.024	-0.046	-0.046	-0.046	0.622
peace	0.006	-0.001	0.018	-0.102	-0.102	-0.102	0.221
peach	0.002	0.001	0.126	0.125	0.125	0.125	0.073
plane	0.002	-0.004	0.01	-0.113	-0.113	-0.113	0.408
reliability	0.002	-0.001	0.025	-0.035	-0.035	-0.035	0.361
sad	-0.004	-0.004	-0.058	-0.069	-0.069	-0.069	0.856
safety	0.003	-0.001	-0.014	-0.048	-0.048	-0.048	0.403
sandstorm	0.001	-0.003	0.148	0.193	0.193	0.193	0.343
shirt	0.001	-0.003	0.038	-0.059	-0.059	-0.059	0.614
shoes	0.001	-0.004	0.082	-0.028	-0.028	-0.028	0.51
sky	0.002	-0.002	-0.152	-0.303	-0.303	-0.303	0.439
sleeping	-0.003	-0.004	-0.026	-0.117	-0.117	-0.117	0.863
socks	0.001	-0.004	0.086	0.001	0.001	0.001	0.465
speed	0.003	0.004	-0.021	-0.007	-0.007	-0.007	0.083
strawberry	-0.001	0.002	-0.001	0.12	0.12	0.12	0.232
sunset	0.002	0.003	0.132	-0.012	-0.012	-0.012	0.137
train	-0.001	-0.002	0.022	0.062	0.062	0.062	0.6
truck	-0.002	-0.004	0.041	0.029	0.029	0.029	0.593
watermelon	-0.001	0.003	-0.06	0.167	0.167	0.167	0.233
working	-0.001	-0.003	-0.017	0.043	0.043	0.043	0.639

Table I.1: Regression coefficients for color space regression models fit to each concept's color-concept associations. L refers to lightness, C refers to chroma, H refers to hue angle and k is a constant.

J Human and GPT-4 Color-Concept Association Distributions

**Color**	**Sorted Position**	**x**	**y**	**Y**	**L***	**a***	**b***
1	50	0.178	0.140	18.42	50.00	28.89	−73.59
2	53	0.174	0.083	4.42	25.00	53.86	−72.28
3	54	0.217	0.136	18.42	50.00	53.86	−72.28
4	55	0.259	0.131	18.42	50.00	78.82	−70.97
5	46	0.187	0.192	18.42	50.00	2.62	−49.93
6	51	0.191	0.130	4.42	25.00	27.58	−48.62
7	52	0.231	0.184	8.42	50.00	27.58	−48.62
8	57	0.255	0.123	4.42	25.00	52.55	−47.32
9	56	0.279	0.176	18.42	50.00	52.55	−47.32
10	61	0.328	0.167	18.42	50.00	77.51	−46.01
11	45	0.224	0.284	48.28	75.00	−23.66	−26.27
12	47	0.208	0.214	4.42	25.00	1.31	−24.97

13	48	0.245	0.254	18.42	50.00	1.31	−24.97
14	49	0.263	0.274	48.28	75.00	1.31	−24.97
15	58	0.286	0.199	4.42	25.00	26.27	−23.66
16	59	0.298	0.241	18.42	50.00	26.27	−23.66
17	60	0.303	0.262	48.28	75.00	26.27	−23.66
18	62	0.369	0.181	4.42	25.00	51.24	−22.35
19	63	0.353	0.226	18.42	50.00	51.24	−22.35
20	64	0.408	0.211	18.42	50.00	76.21	−21.04
21	44	0.238	0.357	72.07	88.00	−49.93	−2.62
22	43	0.253	0.351	18.42	50.00	−24.97	−1.31
23	42	0.269	0.345	48.28	75.00	−24.97	−1.31
24	41	0.275	0.343	72.07	88.00	−24.97	−1.31
25	6	0.313	0.329	0.00	0.00	0.00	0.00
26	5	0.313	0.329	4.42	25.00	0.00	0.00
27	4	0.313	0.329	18.42	50.00	0.00	0.00
28	3	0.313	0.329	48.28	75.00	0.00	0.00
29	1	0.313	0.329	100.00	100.00	0.00	0.00
30	2	0.313	0.329	72.07	88.00	0.00	0.00
31	67	0.410	0.291	4.42	25.00	24.97	1.31
32	68	0.374	0.305	18.42	50.00	24.97	1.31
33	69	0.357	0.312	48.28	75.00	24.97	1.31
34	66	0.434	0.281	18.42	50.00	49.93	2.62
35	65	0.492	0.257	18.42	50.00	74.90	3.93
36	40	0.270	0.433	48.28	75.00	−51.24	22.35
37	39	0.276	0.418	72.07	88.00	−51.24	22.35
38	36	0.308	0.524	4.42	25.00	−26.27	23.66
39	33	0.316	0.444	18.42	50.00	−26.27	23.66
40	32	0.317	0.410	48.28	75.00	−26.27	23.66
41	31	0.317	0.399	72.07	88.00	−26.27	23.66
42	20	0.418	0.450	4.42	25.00	−1.31	24.97
43	17	0.382	0.407	18.42	50.00	−1.31	24.97
44	16	0.364	0.386	48.28	75.00	−1.31	24.97
45	15	0.357	0.379	72.07	88.00	−1.31	24.97
46	10	0.522	0.377	4.42	25.00	23.66	26.27
47	9	0.447	0.370	18.42	50.00	23.66	26.27
48	8	0.410	0.362	48.28	75.00	23.66	26.27
49	7	0.509	0.333	18.42	50.00	48.62	27.58
50	70	0.566	0.299	18.42	50.00	73.59	28.89
51	38	0.297	0.577	18.42	50.00	−52.55	47.32
52	35	0.310	0.503	48.28	75.00	−52.55	47.32

53	34	0.313	0.479	72.07	88.00	−52.55	47.32
54	28	0.368	0.525	18.42	50.00	−27.58	48.62
55	27	0.360	0.471	48.28	75.00	−27.58	48.62
56	26	0.356	0.453	72.07	88.00	−27.58	48.62
57	22	0.437	0.472	18.42	50.00	−2.62	49.93
58	19	0.409	0.439	48.28	75.00	−2.62	49.93
59	18	0.399	0.427	72.07	88.00	−2.62	49.93
60	13	0.502	0.421	18.42	50.00	22.35	51.24
61	12	0.457	0.407	48.28	75.00	22.35	51.24
62	11	0.563	0.373	18.42	50.00	47.32	52.55
63	71	0.618	0.330	18.42	50.00	72.28	53.86
64	37	0.300	0.564	72.07	88.00	−78.82	70.97
65	29	0.343	0.561	48.28	75.00	−53.86	72.28
66	30	0.345	0.532	72.07	88.00	−53.86	72.28
67	24	0.394	0.521	48.28	75.00	−28.89	73.59
68	25	0.389	0.500	72.07	88.00	−28.89	73.59
69	23	0.444	0.481	48.28	75.00	−3.93	74.90
70	21	0.432	0.467	72.07	88.00	−3.93	74.90
71	14	0.492	0.443	48.28	75.00	21.04	76.21

Table J.1: Coordinates for the UW-71 colors in CIE 1931 xyY space and CIELAB color space. The white point used to convert between CIE 1931 xyY and CIELAB space was CIE Illuminant D65 (x = 0.313, y = 0.329, Y = 100). The 'Sorted Position' column indicates the index of the color when the colors are sorted by hue angle and chroma.

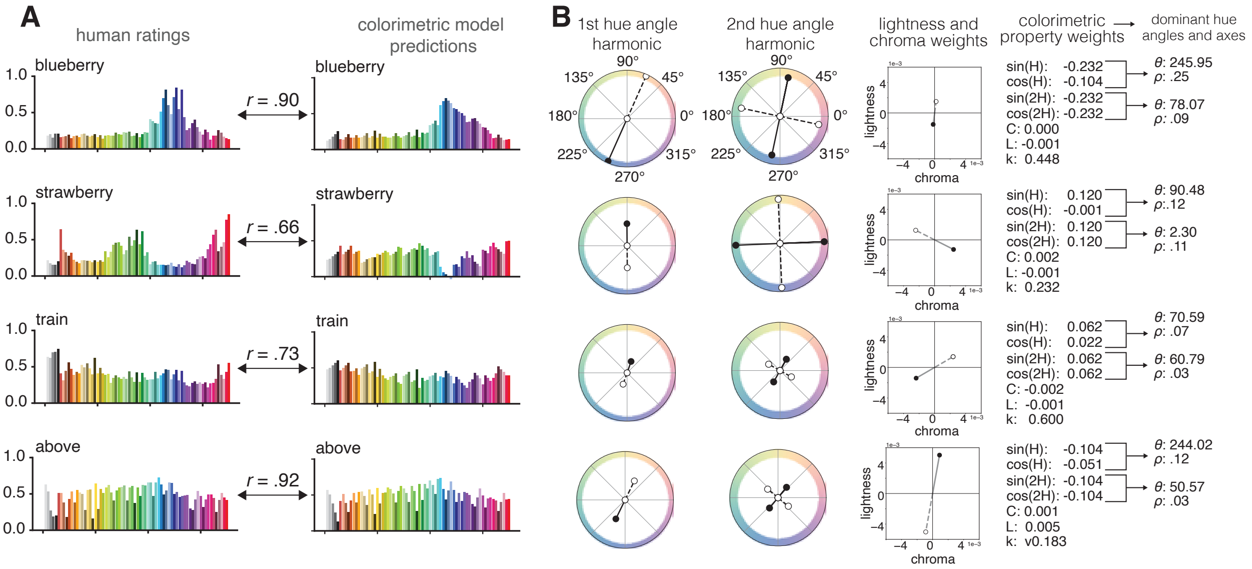

Figure I.1: (A) Mean human color-concept association ratings between the concept and the `UW-71` colors and predicted associations from color space regression models. (B) Visualization of the coefficients of the color space regression models. From left to right, dominant hue angle, dominant hue axis, lightness and chroma, and numeric representations of the coefficients. The weights on the sines and cosines of the hue angle and the double hue angle are used to compute the dominant hues and axes visualized in the middle columns.

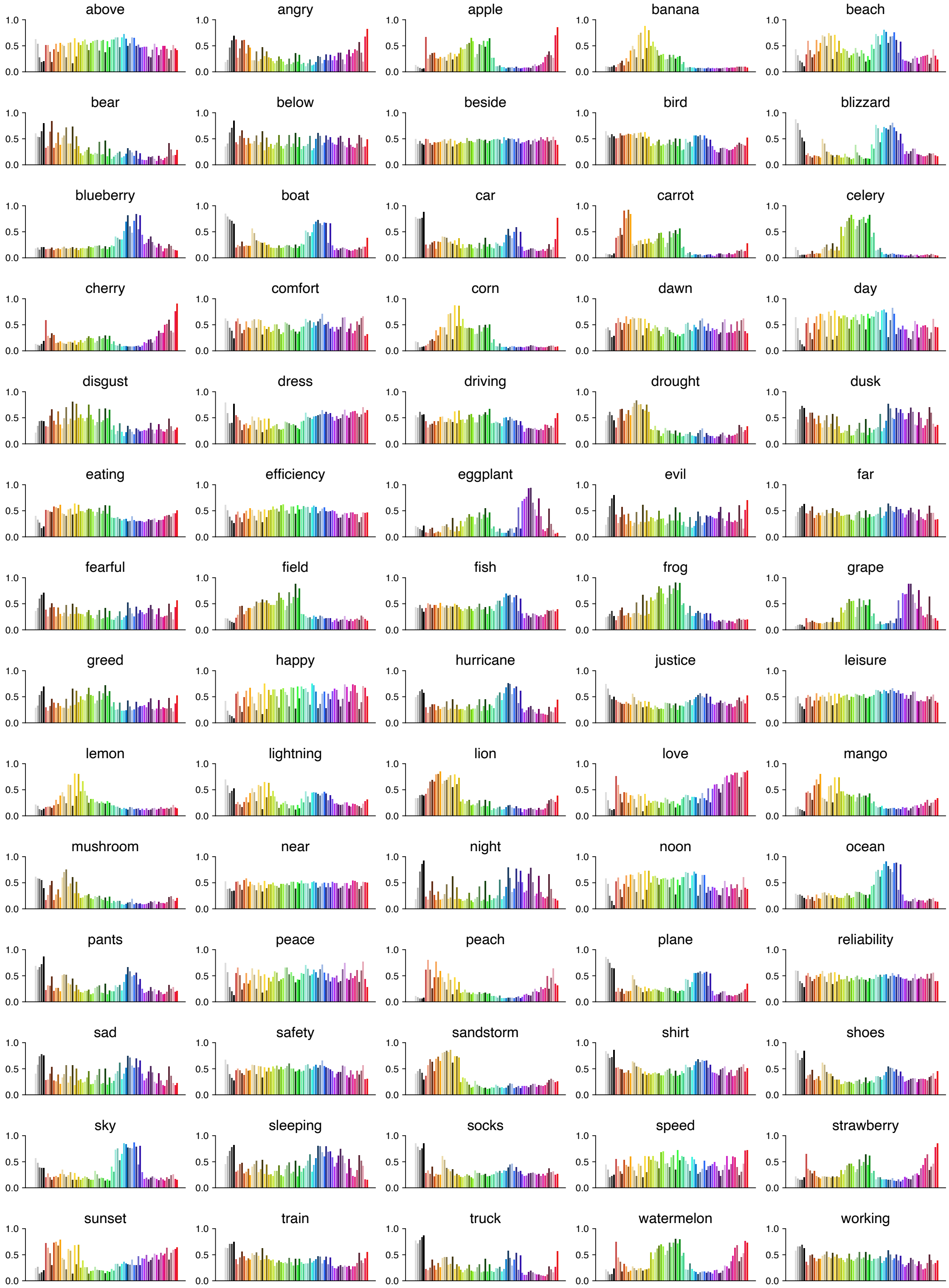

Figure J.1: Mean **human** color-concept association ratings for all 70 concepts across the `UW-71` colors.

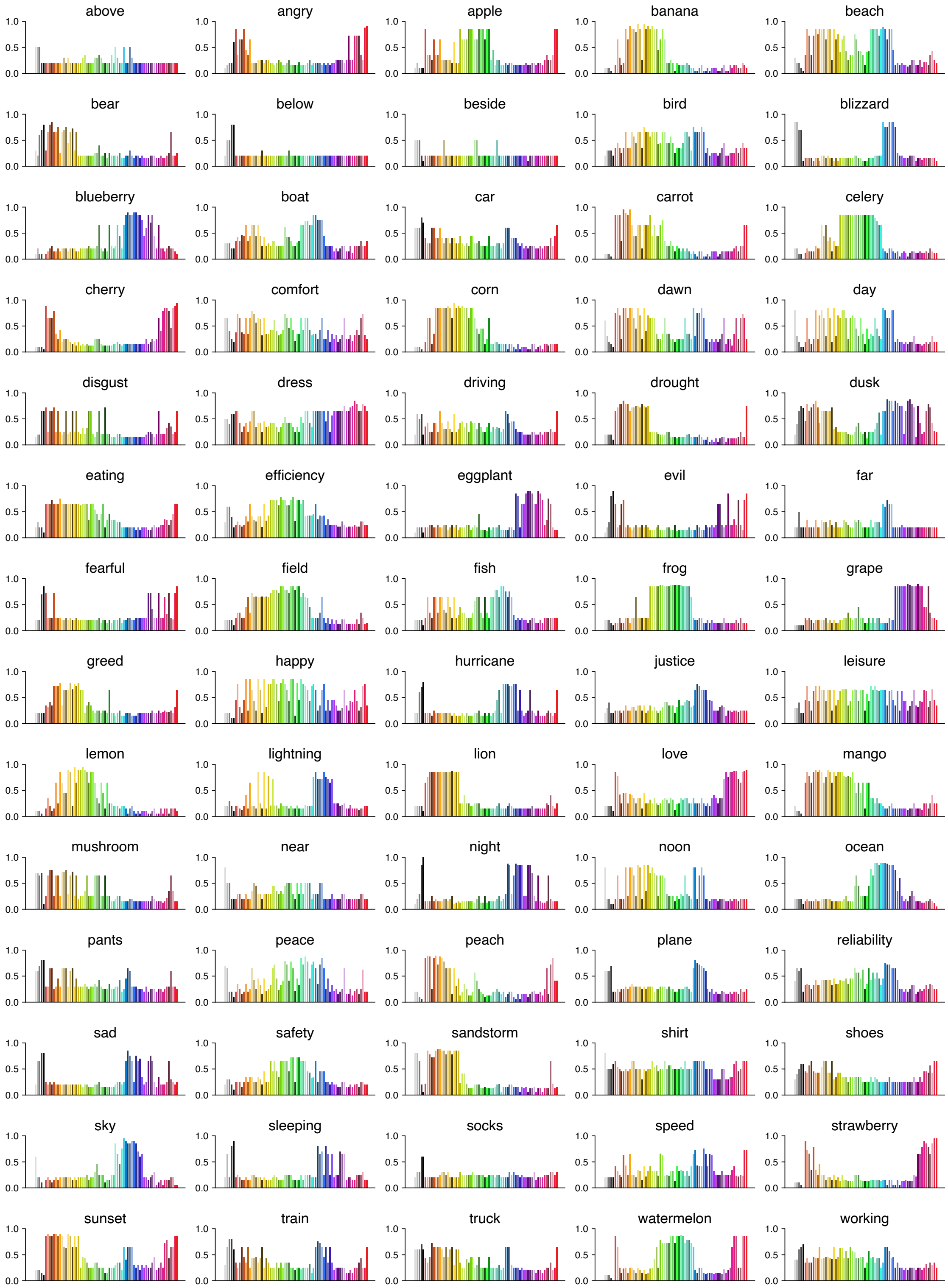

Figure J.2: **GPT-4** color concept associations for all 70 concepts across the UW-71 colors from **Experiment 1** in the No Anchoring condition.

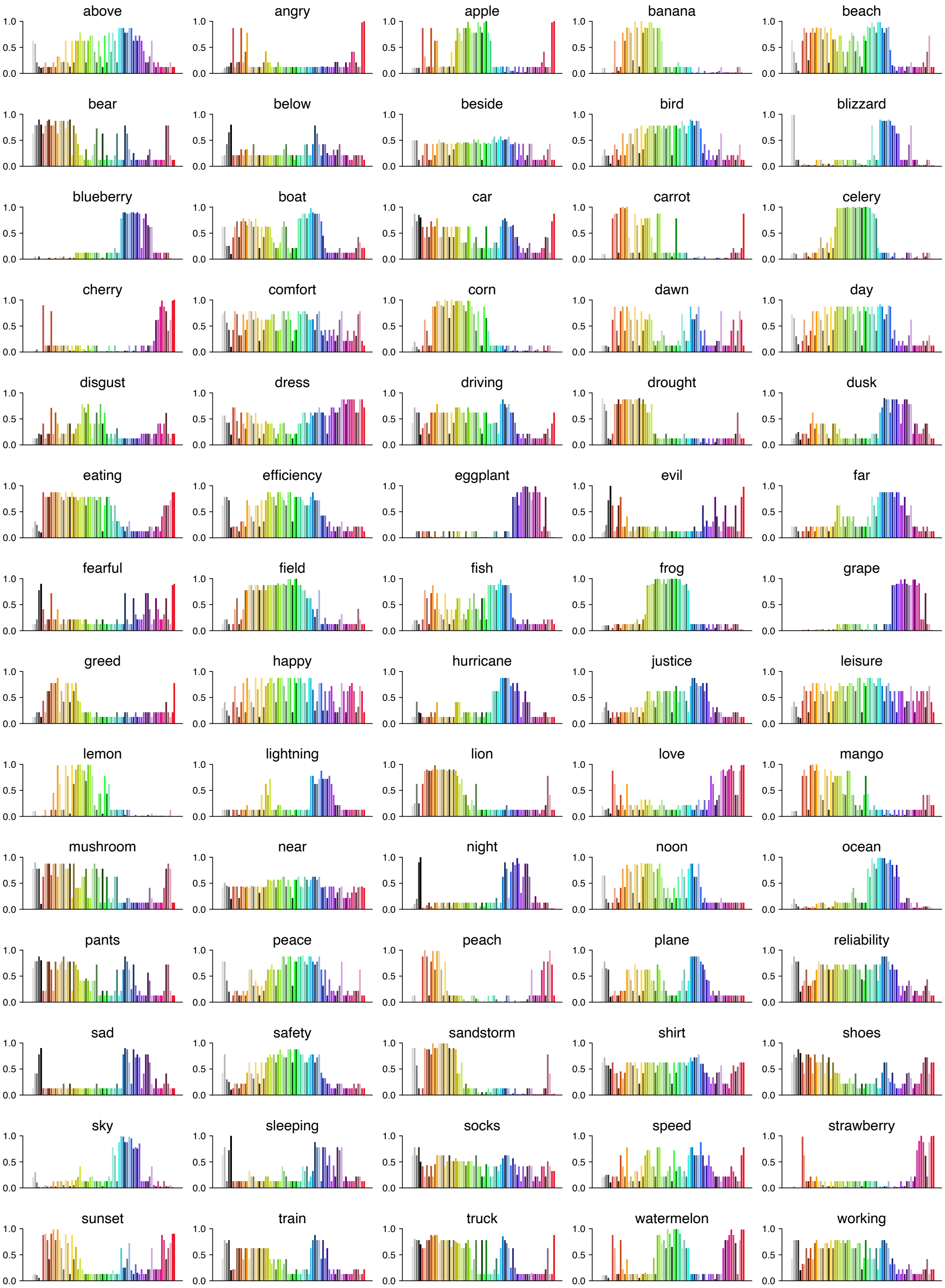

Figure J.3: **GPT-4** color concept associations for all 70 concepts across the `UW-71` colors from **Experiment 1** in the Anchoring condition.

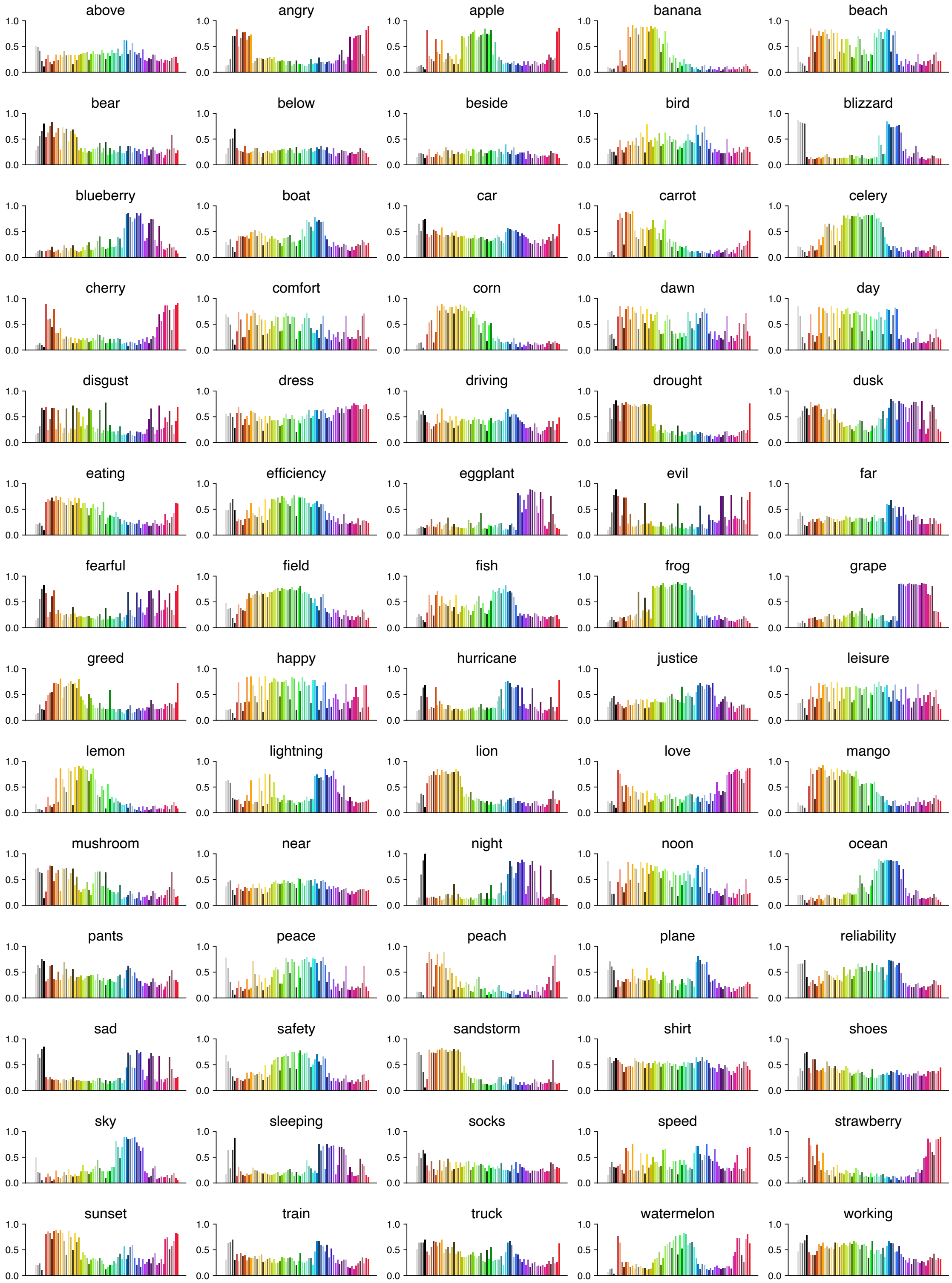

Figure J.4: **GPT-4** color concept associations for all 70 concepts across the `UW-71` colors from **Experiment 1** in the Multiple Ratings condition.

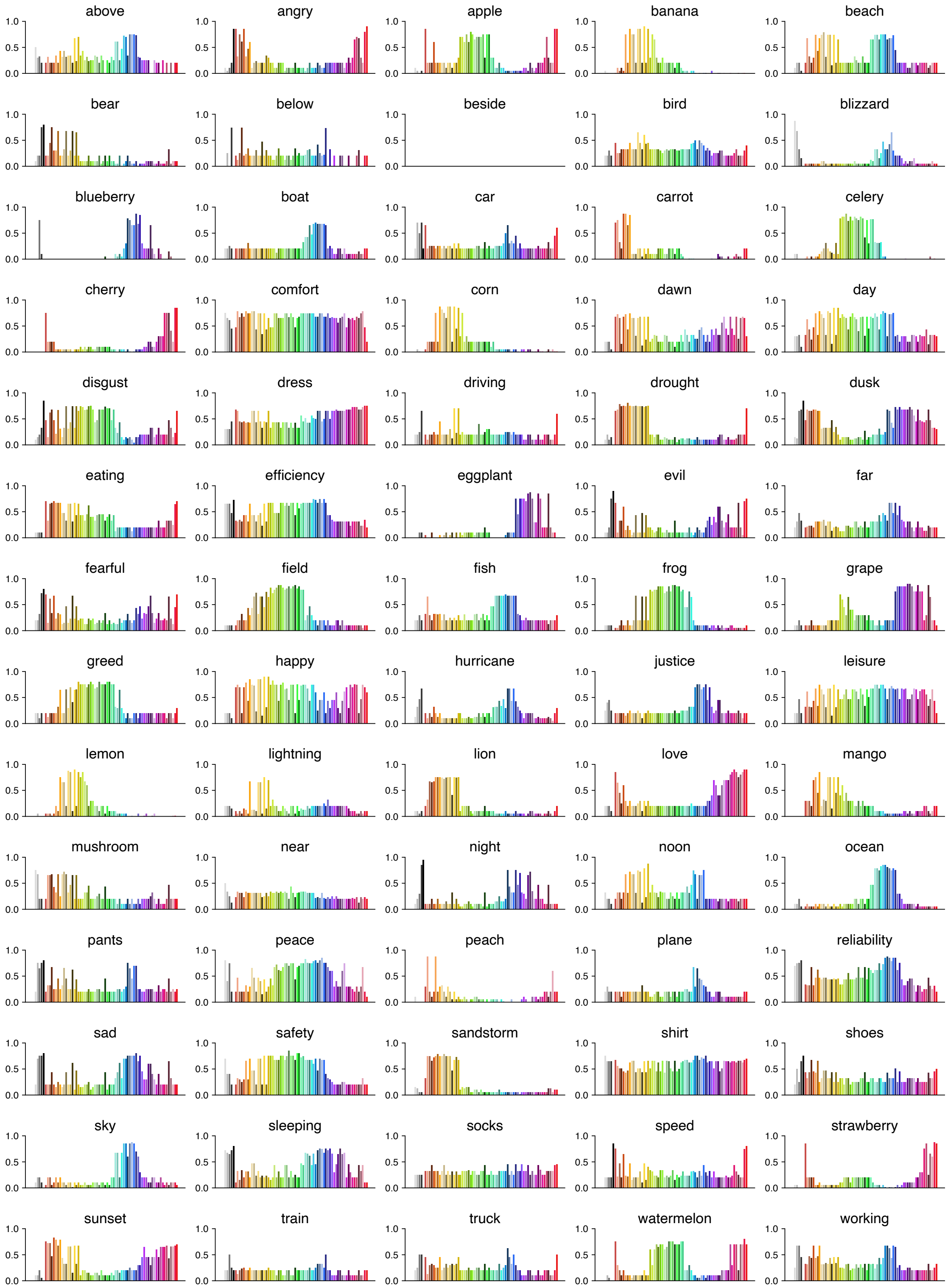

Figure J.5: **GPT-4** color concept associations for all 70 concepts across the UW-71 colors from **Experiment 2** in the Color Patches condition.

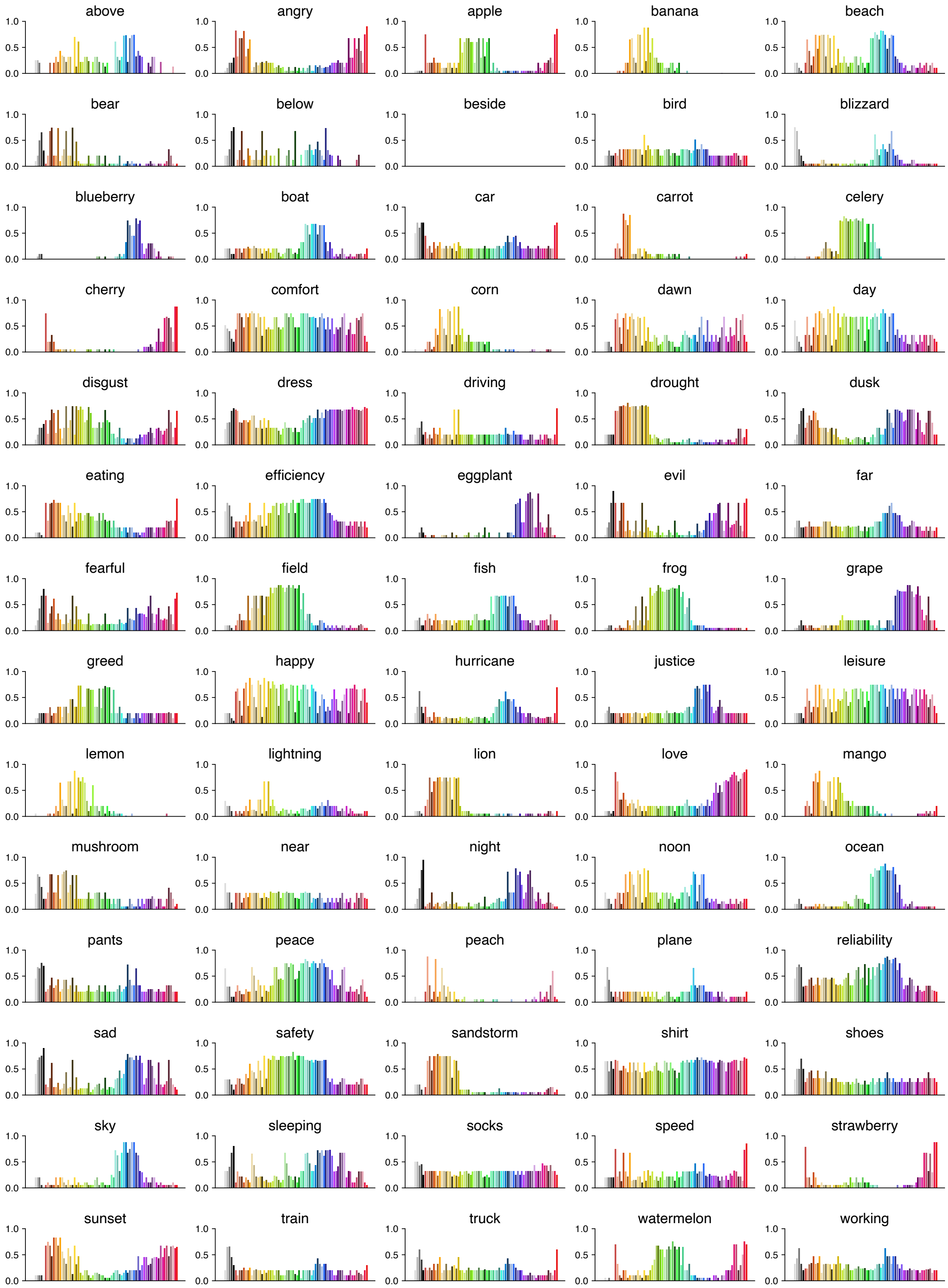

Figure J.6: **GPT-4** color concept associations for all 70 concepts across the `UW-71` colors from **Experiment 2** in the Patches and Hex codes condition.

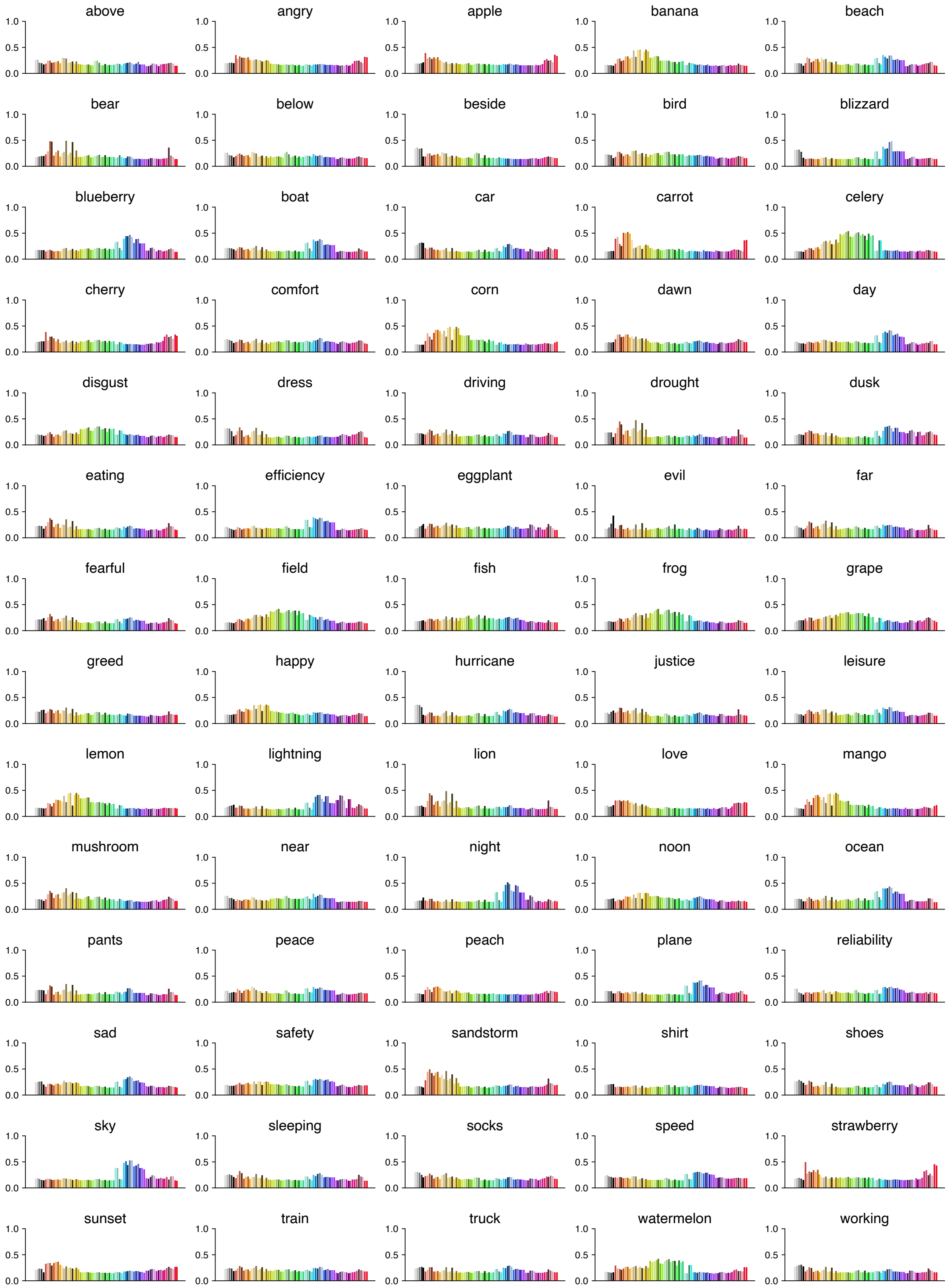

Figure J.7: **GPT-4** color concept associations from **Experiment 2** when using the method from Rathore et al. (2020) [5], which leverages information from Google images, for all 70 concepts across the `UW-71` colors.

Figure J.8: Correlations between human color-concept association ratings and estimated associations for all GPT-4 prompt methods in Experiments 1 and 2.

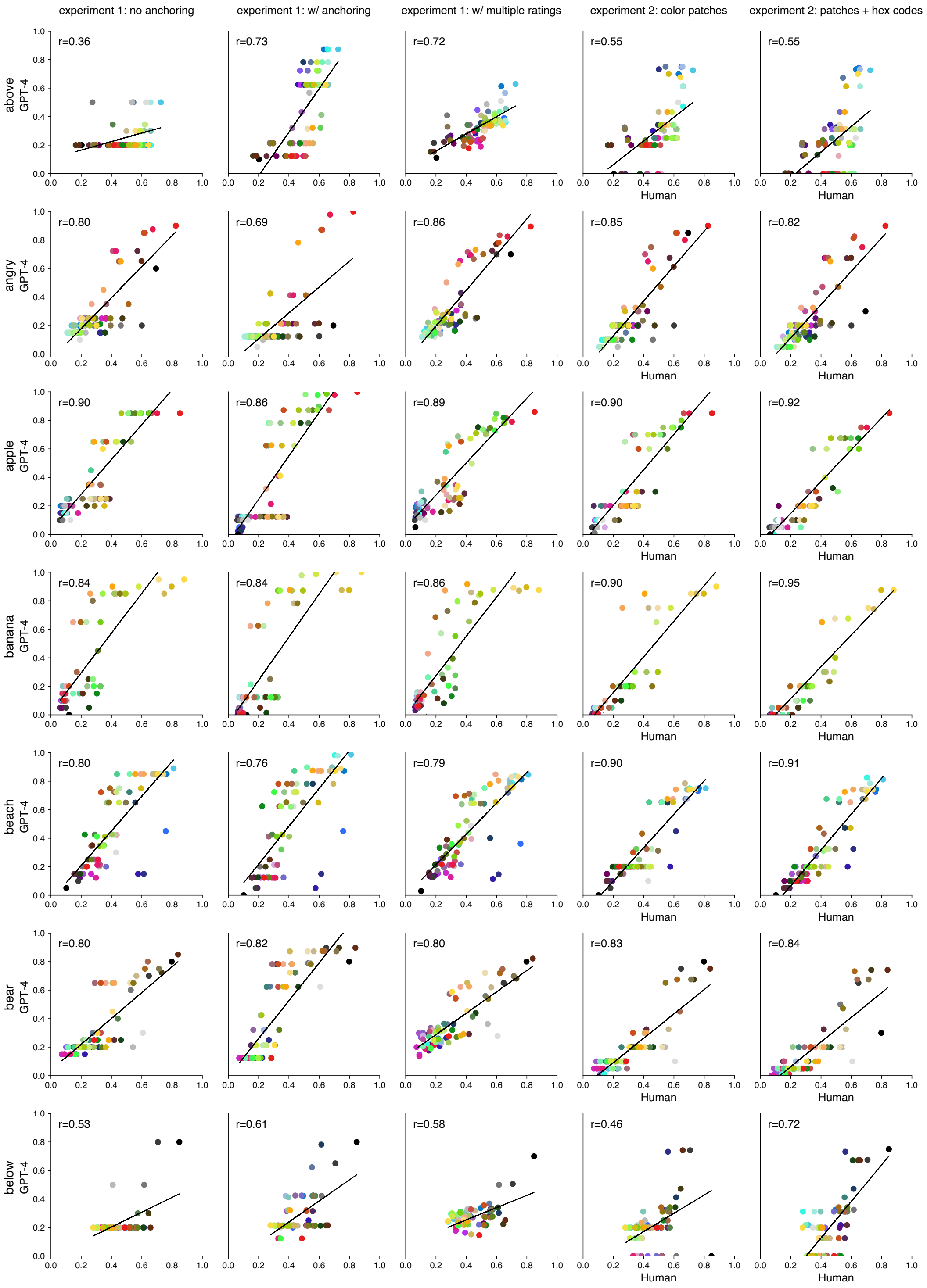

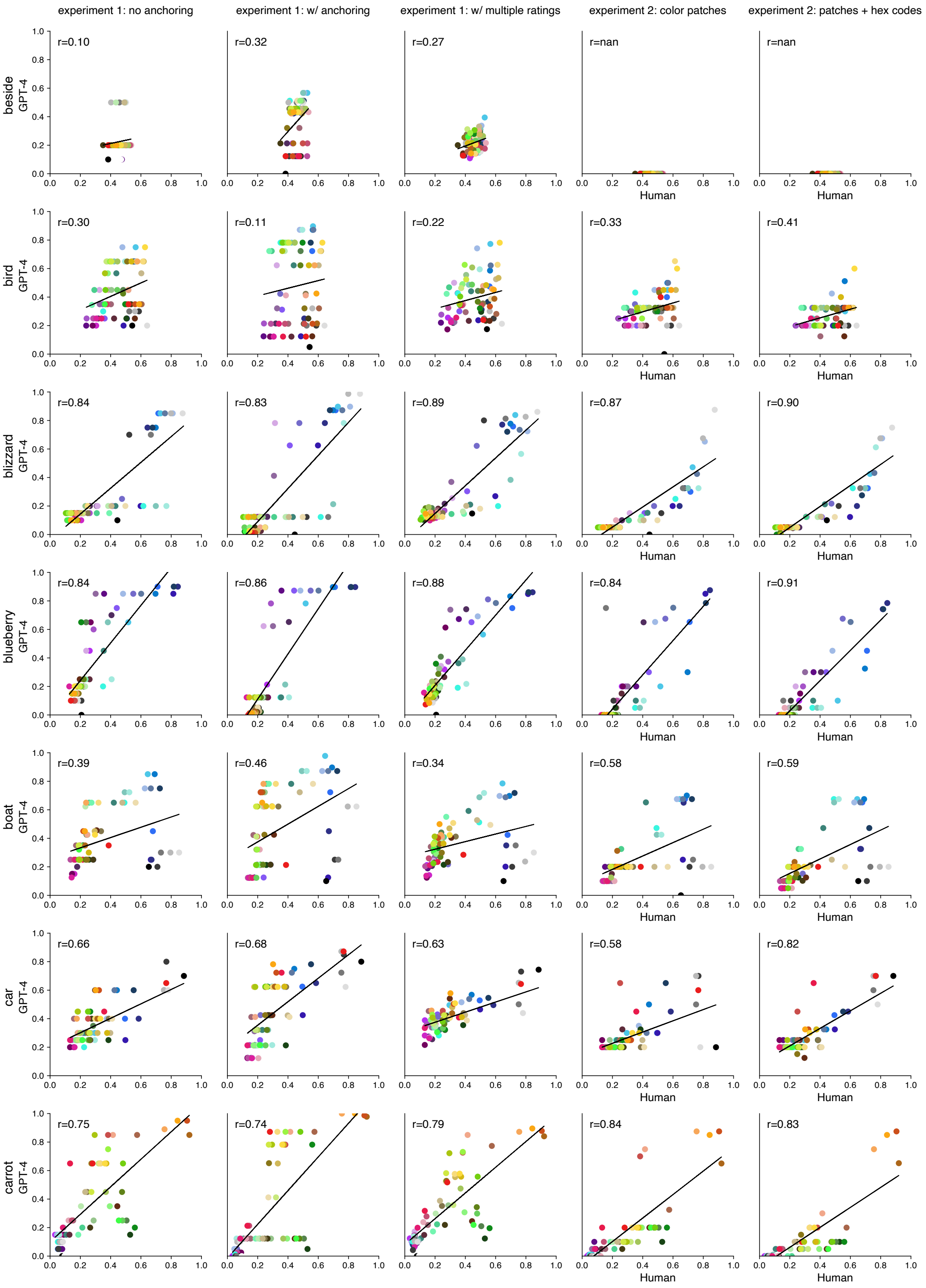

experiment 1: no anchoring
experiment 1: w/ anchoring
experiment 1: w/ multiple ratings
experiment 2: color patches
experiment 2: patches + hex codes
beside GPT-4
r=0.10
r=0.32
r=0.27
r=nan
r=nan
bird GPT-4
r=0.30
r=0.11
r=0.22
r=0.33
r=0.41
blizzard GPT-4
r=0.84
r=0.83
r=0.89
r=0.87
r=0.90
blueberry GPT-4
r=0.84
r=0.86
r=0.88
r=0.84
r=0.91
boat GPT-4
r=0.39
r=0.46
r=0.34
r=0.58
r=0.59
car GPT-4
r=0.66
r=0.68
r=0.63
r=0.58
r=0.82
carrot GPT-4
r=0.75
r=0.74
r=0.79
r=0.84
r=0.83
Human
0.0
0.2
0.4
0.6
0.8
1.0

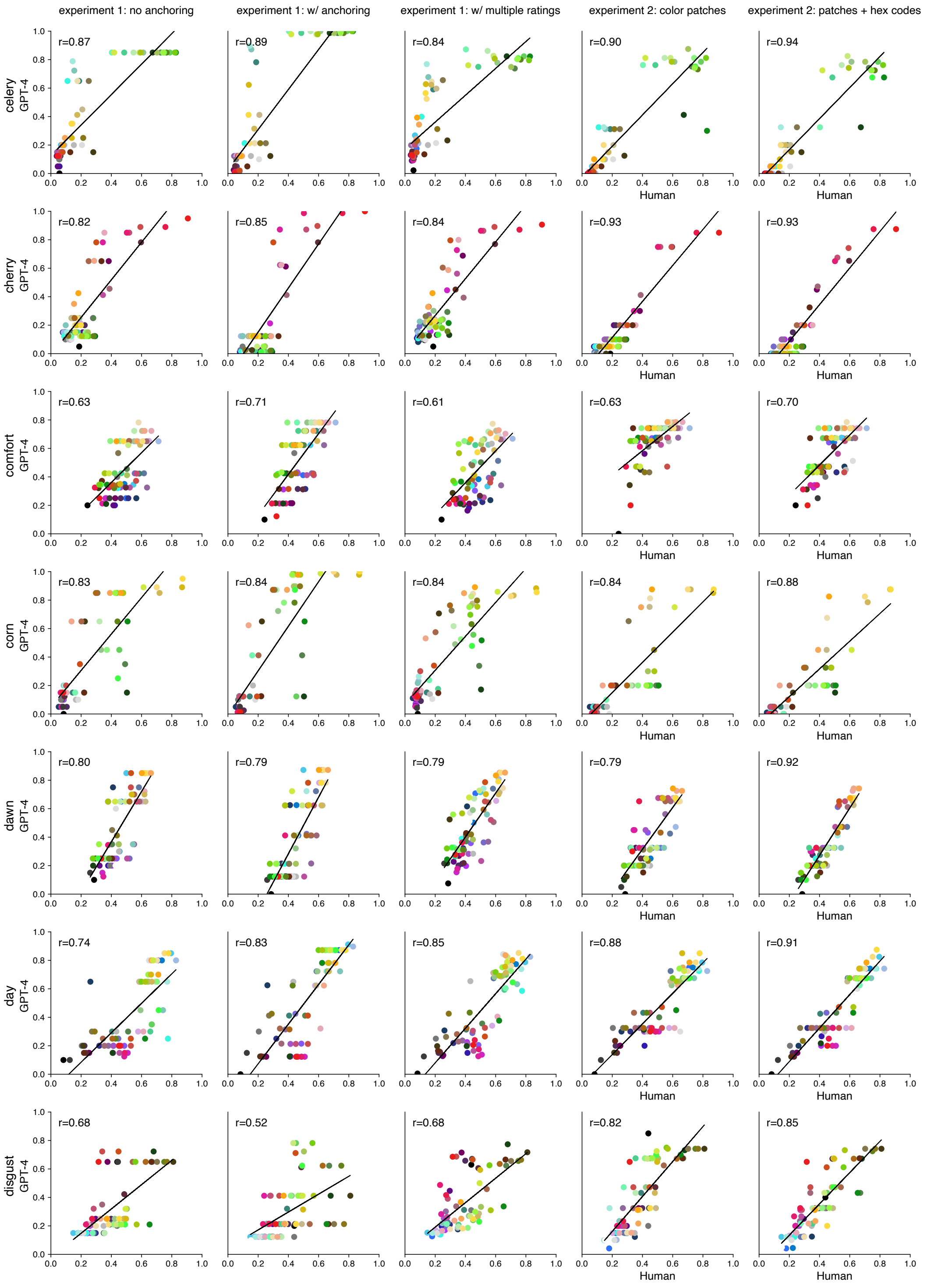
experiment 1: no anchoring
experiment 1: w/ anchoring
experiment 1: w/ multiple ratings
experiment 2: color patches
experiment 2: patches + hex codes
celery
GPT-4
r=0.87
r=0.89
r=0.84
r=0.90
r=0.94
cherry
GPT-4
r=0.82
r=0.85
r=0.84
r=0.93
r=0.93
comfort
GPT-4
r=0.63
r=0.71
r=0.61
r=0.63
r=0.70
corn
GPT-4
r=0.83
r=0.84
r=0.84
r=0.84
r=0.88
dawn
GPT-4
r=0.80
r=0.79
r=0.79
r=0.79
r=0.92
day
GPT-4
r=0.74
r=0.83
r=0.85
r=0.88
r=0.91
disgust
GPT-4
r=0.68
r=0.52
r=0.68
r=0.82
r=0.85
Human

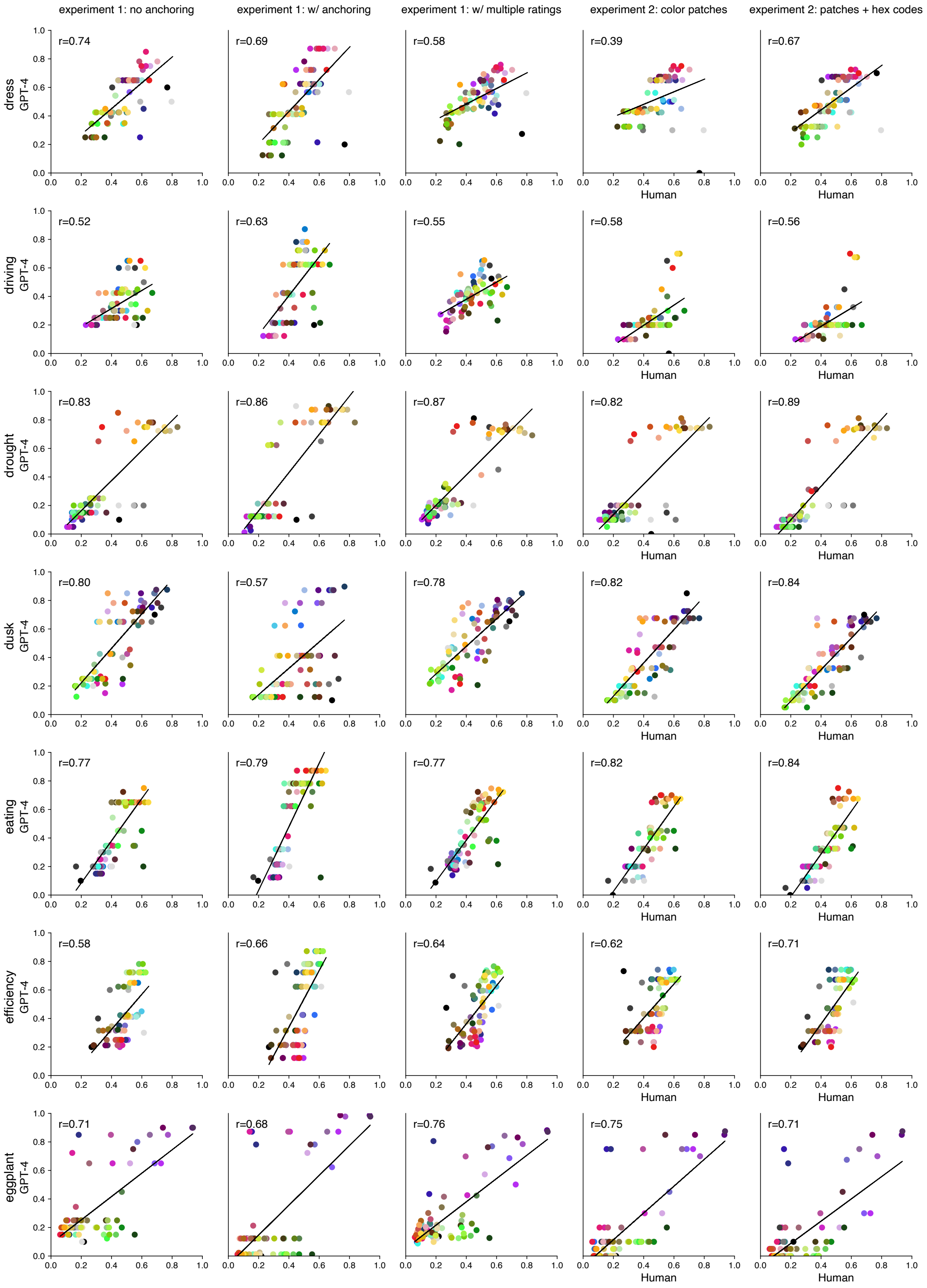
experiment 1: no anchoring
experiment 1: w/ anchoring
experiment 1: w/ multiple ratings
experiment 2: color patches
experiment 2: patches + hex codes
dress GPT-4
r=0.74
r=0.69
r=0.58
r=0.39
r=0.67
driving GPT-4
r=0.52
r=0.63
r=0.55
r=0.58
r=0.56
drought GPT-4
r=0.83
r=0.86
r=0.87
r=0.82
r=0.89
dusk GPT-4
r=0.80
r=0.57
r=0.78
r=0.82
r=0.84
eating GPT-4
r=0.77
r=0.79
r=0.77
r=0.82
r=0.84
efficiency GPT-4
r=0.58
r=0.66
r=0.64
r=0.62
r=0.71
eggplant GPT-4
r=0.71
r=0.68
r=0.76
r=0.75
r=0.71
Human

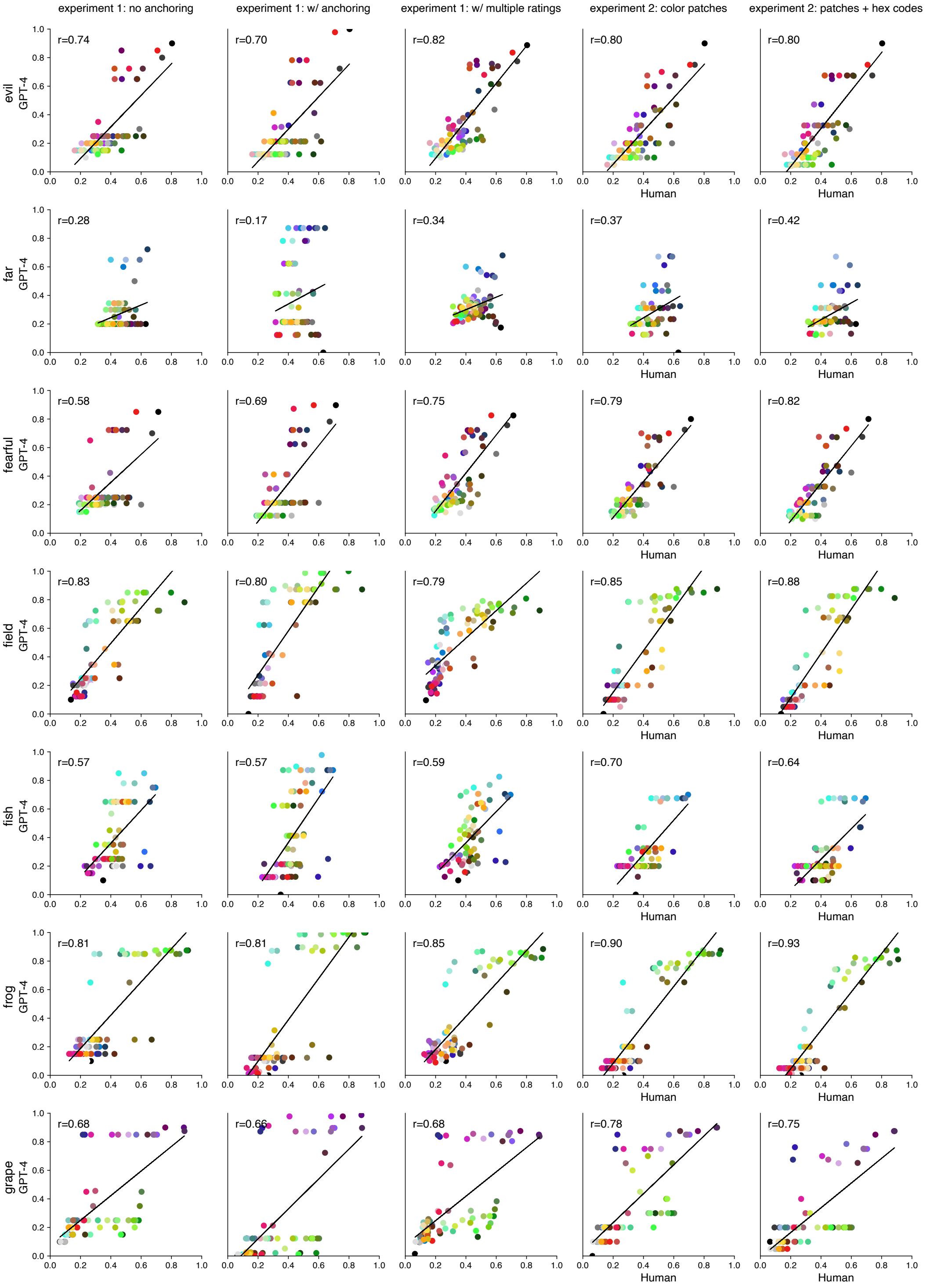
experiment 1: no anchoring
experiment 1: w/ anchoring
experiment 1: w/ multiple ratings
experiment 2: color patches
experiment 2: patches + hex codes
evil
far
fearful
field
fish
frog
grape
GPT-4
Human
r=0.74
r=0.70
r=0.82
r=0.80
r=0.80
r=0.28
r=0.17
r=0.34
r=0.37
r=0.42
r=0.58
r=0.69
r=0.75
r=0.79
r=0.82
r=0.83
r=0.80
r=0.79
r=0.85
r=0.88
r=0.57
r=0.57
r=0.59
r=0.70
r=0.64
r=0.81
r=0.81
r=0.85
r=0.90
r=0.93
r=0.68
r=0.66
r=0.68
r=0.78
r=0.75

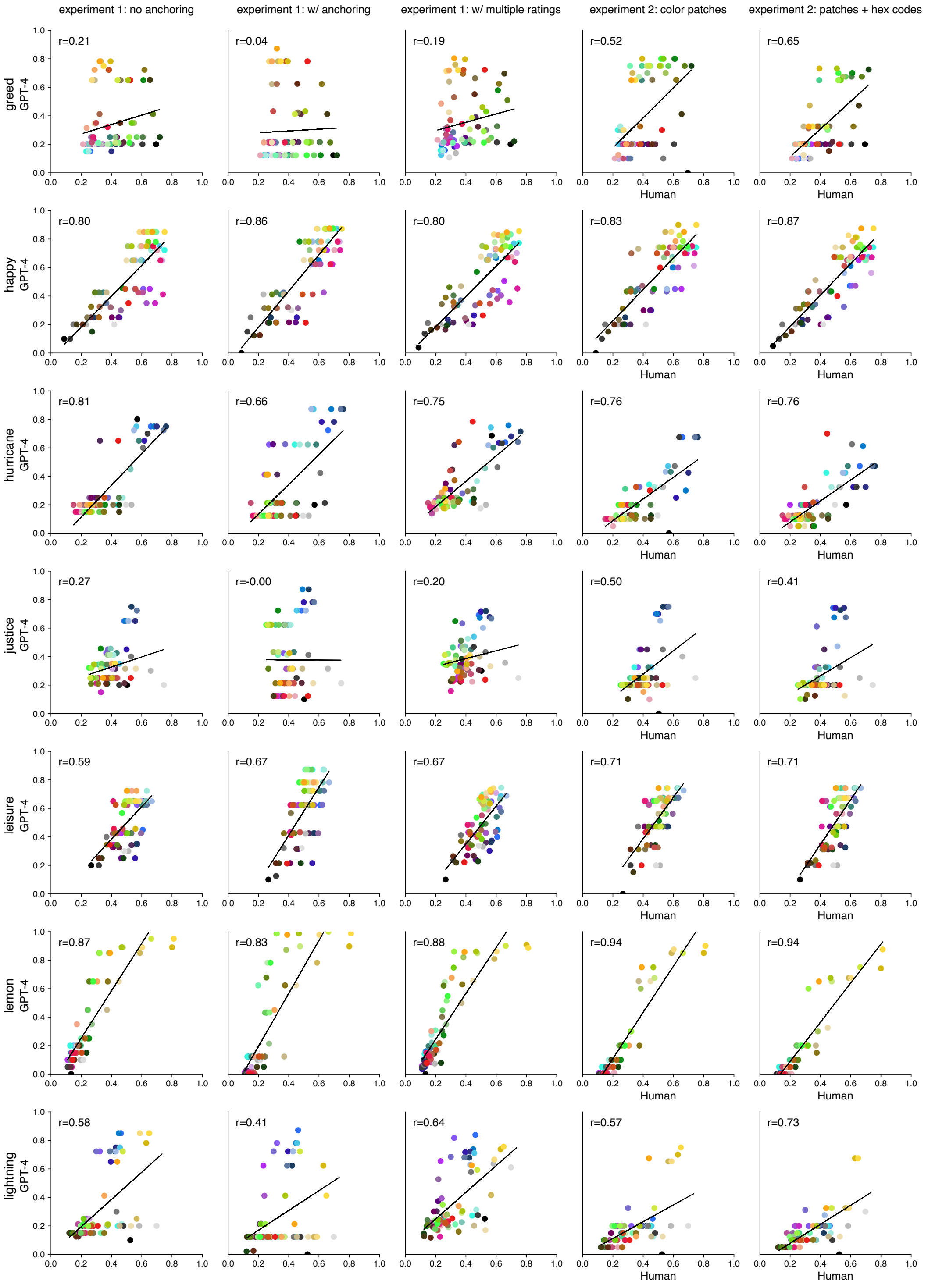

experiment 1: no anchoring
experiment 1: w/ anchoring
experiment 1: w/ multiple ratings
experiment 2: color patches
experiment 2: patches + hex codes
greed
happy
hurricane
justice
leisure
lemon
lightning
GPT-4
Human
r=0.21
r=0.04
r=0.19
r=0.52
r=0.65
r=0.80
r=0.86
r=0.80
r=0.83
r=0.87
r=0.81
r=0.66
r=0.75
r=0.76
r=0.76
r=0.27
r=-0.00
r=0.20
r=0.50
r=0.41
r=0.59
r=0.67
r=0.67
r=0.71
r=0.71
r=0.87
r=0.83
r=0.88
r=0.94
r=0.94
r=0.58
r=0.41
r=0.64
r=0.57
r=0.73

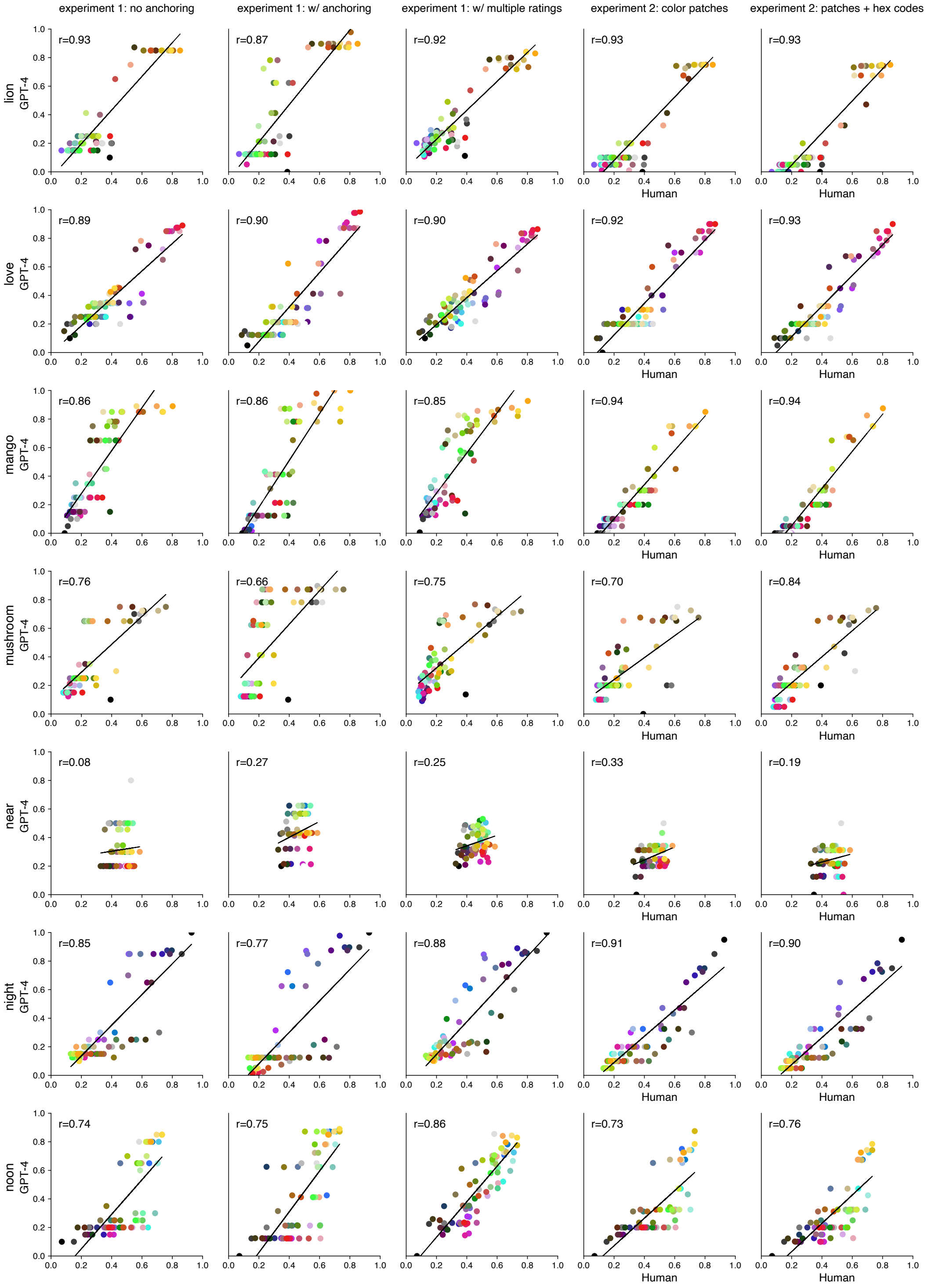

experiment 1: no anchoring
experiment 1: w/ anchoring
experiment 1: w/ multiple ratings
experiment 2: color patches
experiment 2: patches + hex codes
lion
love
mango
mushroom
near
night
noon
GPT-4
Human
r=0.93
r=0.87
r=0.92
r=0.93
r=0.93
r=0.89
r=0.90
r=0.90
r=0.92
r=0.93
r=0.86
r=0.86
r=0.85
r=0.94
r=0.94
r=0.76
r=0.66
r=0.75
r=0.70
r=0.84
r=0.08
r=0.27
r=0.25
r=0.33
r=0.19
r=0.85
r=0.77
r=0.88
r=0.91
r=0.90
r=0.74
r=0.75
r=0.86
r=0.73
r=0.76

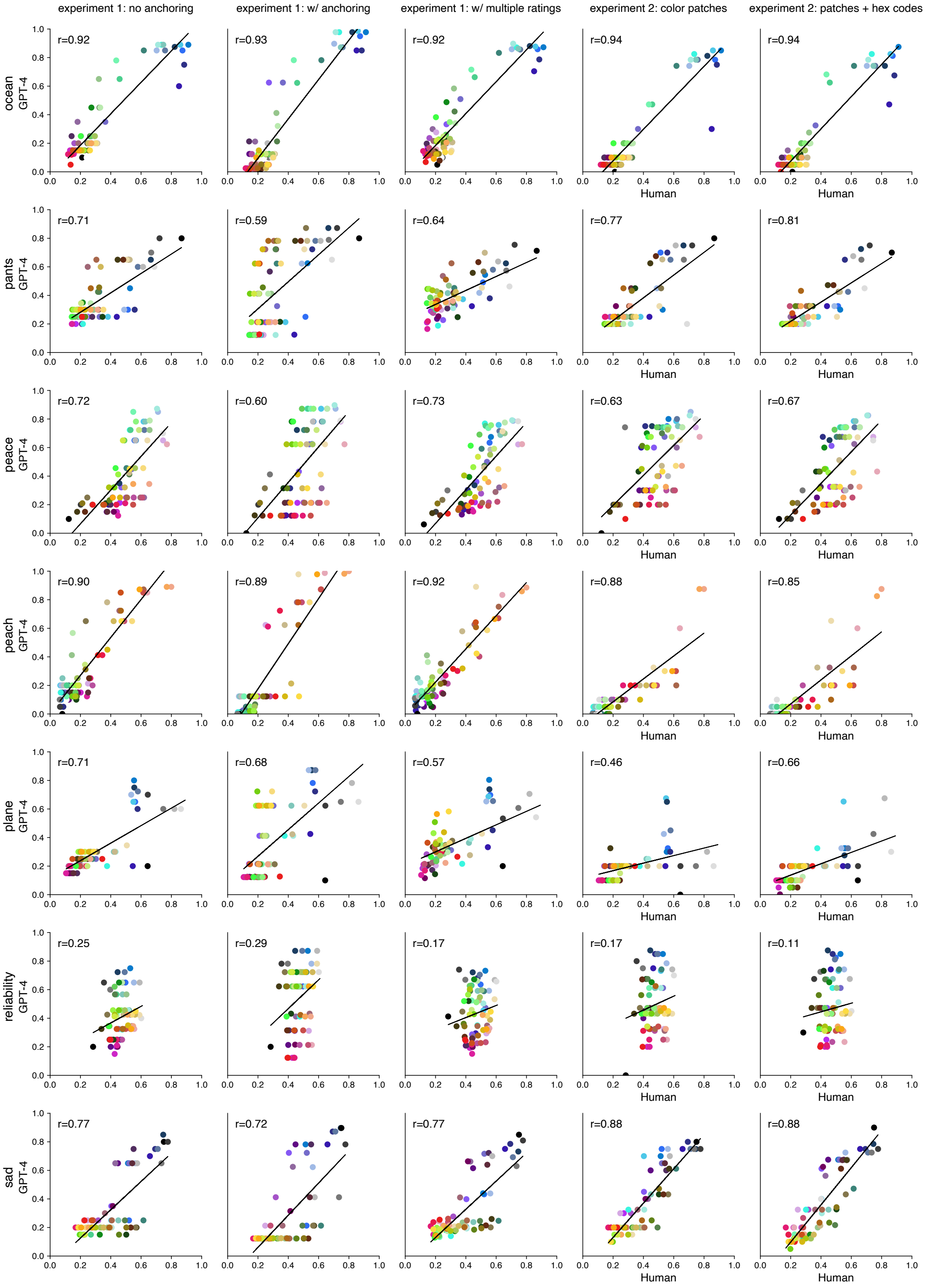

experiment 1: no anchoring
experiment 1: w/ anchoring
experiment 1: w/ multiple ratings
experiment 2: color patches
experiment 2: patches + hex codes
ocean GPT-4
r=0.92
r=0.93
r=0.92
r=0.94
r=0.94
pants GPT-4
r=0.71
r=0.59
r=0.64
r=0.77
r=0.81
peace GPT-4
r=0.72
r=0.60
r=0.73
r=0.63
r=0.67
peach GPT-4
r=0.90
r=0.89
r=0.92
r=0.88
r=0.85
plane GPT-4
r=0.71
r=0.68
r=0.57
r=0.46
r=0.66
reliability GPT-4
r=0.25
r=0.29
r=0.17
r=0.17
r=0.11
sad GPT-4
r=0.77
r=0.72
r=0.77
r=0.88
r=0.88
Human

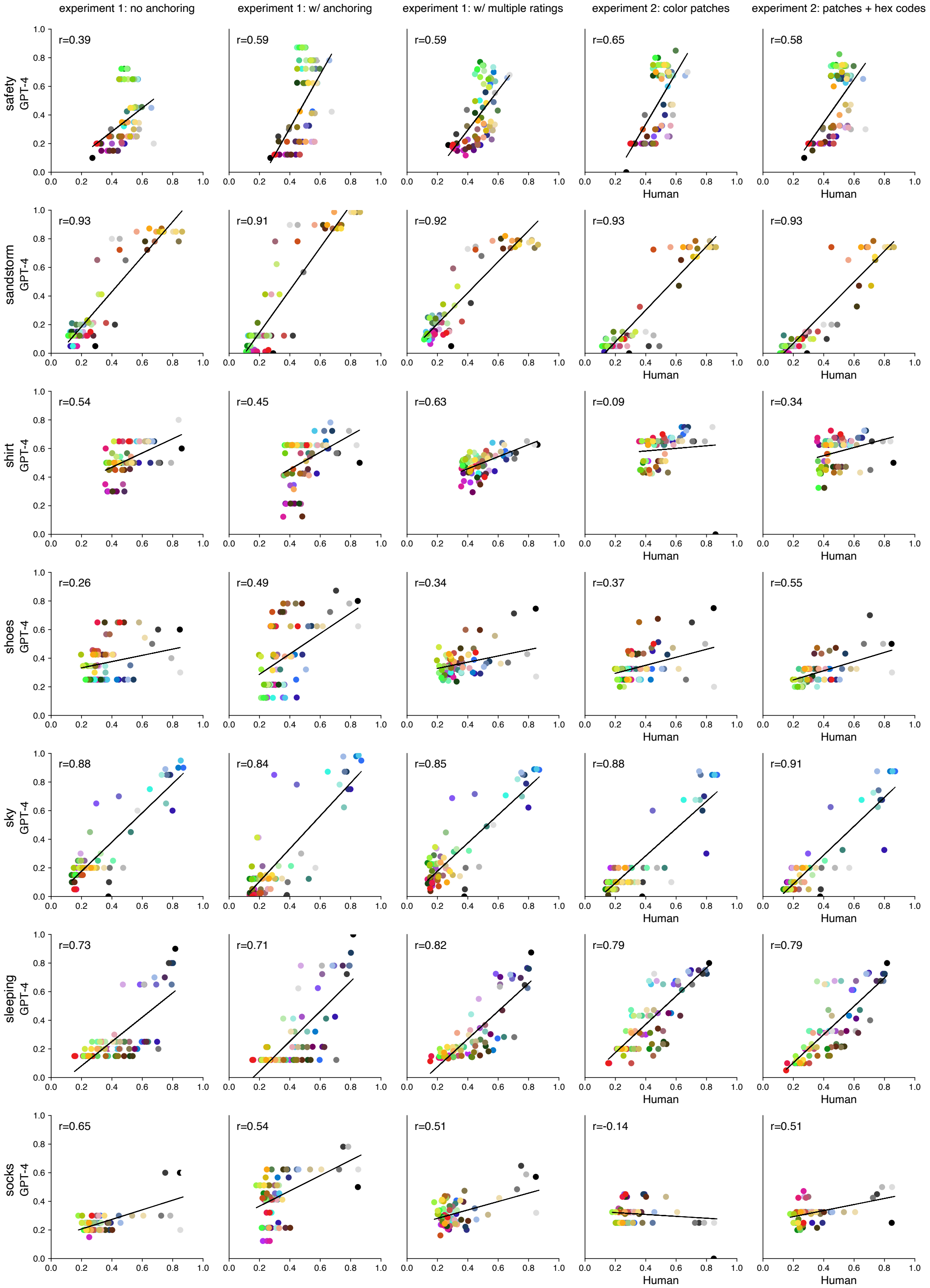

experiment 1: no anchoring
experiment 1: w/ anchoring
experiment 1: w/ multiple ratings
experiment 2: color patches
experiment 2: patches + hex codes
safety
sandstorm
shirt
shoes
sky
sleeping
socks
GPT-4
Human
r=0.39
r=0.59
r=0.59
r=0.65
r=0.58
r=0.93
r=0.91
r=0.92
r=0.93
r=0.93
r=0.54
r=0.45
r=0.63
r=0.09
r=0.34
r=0.26
r=0.49
r=0.34
r=0.37
r=0.55
r=0.88
r=0.84
r=0.85
r=0.88
r=0.91
r=0.73
r=0.71
r=0.82
r=0.79
r=0.79
r=0.65
r=0.54
r=0.51
r=-0.14
r=0.51

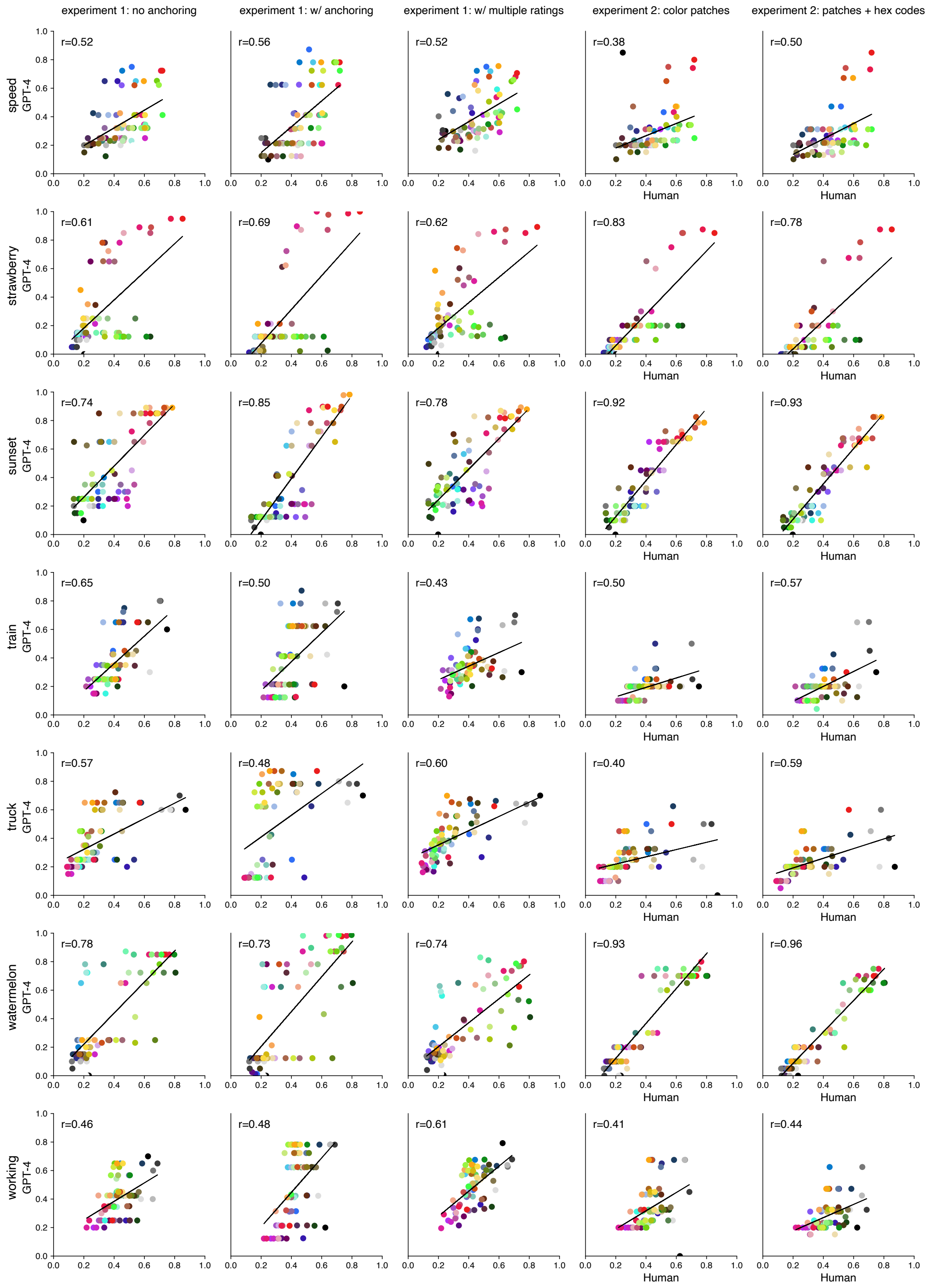
experiment 1: no anchoring
experiment 1: w/ anchoring
experiment 1: w/ multiple ratings
experiment 2: color patches
experiment 2: patches + hex codes
speed
GPT-4
r=0.52
r=0.56
r=0.52
r=0.38
r=0.50
strawberry
GPT-4
r=0.61
r=0.69
r=0.62
r=0.83
r=0.78
sunset
GPT-4
r=0.74
r=0.85
r=0.78
r=0.92
r=0.93
train
GPT-4
r=0.65
r=0.50
r=0.43
r=0.50
r=0.57
truck
GPT-4
r=0.57
r=0.48
r=0.60
r=0.40
r=0.59
watermelon
GPT-4
r=0.78
r=0.73
r=0.74
r=0.93
r=0.96
working
GPT-4
r=0.46
r=0.48
r=0.61
r=0.41
r=0.44
Human

References

[1] Kushin Mukherjee, Brian Yin, Brianne E Sherman, Laurent Lessard, and Karen B Schloss. Context matters: A theory of semantic discriminability for perceptual encoding systems. *IEEE Transactions on Visualization and Computer Graphics*, 28(1):697–706, 2022.

[2] Karen B Schloss, L Lessard, C S Walmsley, and K Foley. Color inference in visual communication: the meaning of colors in recycling. *Cognitive Research: Principles and Implications*, 3(1):5, 2018.

[3] Karen B Schloss, Zachary Leggon, and Laurent Lessard. Semantic discriminability for visual communication. *IEEE Transactions on Visualization and Computer Graphics*, 27(2):1022–1031, 2021.

[4] Melissa A Schoenlein, Johnny Campos, Kevin J Lande, Laurent Lessard, and Karen B Schloss. Unifying effects of direct and relational associations for visual communication. *IEEE Transactions on Visualization and Computer Graphics*, 29(1):385–395, 2023.

[5] Ragini Rathore, Zachary Leggon, Laurent Lessard, and Karen B Schloss. Estimating color-concept associations from image statistics. *IEEE transactions on visualization and computer graphics*, 26(1):1226–1235, 2019.

[6] C Alejandro Parraga and Arash Akbarinia. Nice: A computational solution to close the gap from colour perception to colour categorization. *PloS one*, 11(3):e0149538, 2016.

[7] James Munkres. Algorithms for the assignment and transportation problems. *Journal of the society for industrial and applied mathematics*, 5(1):32–38, 1957.

[8] Harold W Kuhn. The hungarian method for the assignment problem. *Naval research logistics quarterly*, 2(1-2):83–97, 1955.

[9] Rainer Burkard, Mauro Dell'Amico, and Silvano Martello. *Assignment problems: revised reprint*. SIAM, 2012.

[10] Anya C Hurlbert and Yazhu Ling. Biological components of sex differences in color preference. *Current biology*, 17(16):R623–R625, 2007.

[11] Yazhu Ling and Anya C Hurlbert. A new model for color preference: Universality and individuality. In *Final Program and Proceedings-IS and T/SID Color Imaging Conference*. Newcastle University, 2007.

[12] Karen B Schloss, Laurent Lessard, Chris Racey, and Anya C Hurlbert. Modeling color preference using color space metrics. *Vision Research*, 151:99–116, 2018.
